# Characterization of in vitro stability for two processive endoglucanases as exogenous fibre biocatalysts in pig nutrition

**DOI:** 10.1038/s41598-022-13124-1

**Published:** 2022-06-01

**Authors:** Laurence Cheng, Weijun Wang, Ming Z. Fan

**Affiliations:** 1 Department of Animal Biosciences, University of Guelph, Guelph, ON N1G 2W1 Canada; 2 One Health Institute, University of Guelph, Guelph, ON N1G 2W1 Canada; 3 Present Address: Canadian Food Inspection Agency (CFIA) - Ontario Operation, Guelph, ON N1G 2W1 Canada

**Keywords:** Biochemistry, Biotechnology, Physiology, Gastroenterology

## Abstract

Development of highly efficacious exogenous fibre degradation enzymes can enhance efficiency of dietary fibre utilization and sustainability of global pork production. The objectives of this study were to investigate in vitro stability for two processive endoglucanases, referred to as GH5-tCel5A1 and GH5-p4818Cel5_2A that were overexpressed in CLEARCOLIBL21(DE3). Three-dimensional models predicted presence of Cys residues on the catalytic site surfaces of GH5-tCel5A1 and GH5-p4818Cel5_2A; and time course experimental results shown that both cellulases were susceptible to auto-oxidation by airborne O_2_ and were unstable. Furthermore, we examined these endoglucanases’ stability under the mimicked in vitro porcine gastric and the small intestinal pH and proteases’ conditions. Eadie-Hofstee inhibition kinetic analyses showed that GH5-tCel5A1 and GH5-p4818Cel5_2A respectively lost 18 and 68% of their initial activities after 2-h incubations under the gastric conditions and then lost more than 90% of their initial activities after 2–3 h of incubations under the small intestinal conditions. Therefore, further enzyme protein engineering to improve resistance and alternatively post-fermentation enzyme processing such as coating to bypass the gastric-small intestinal environment will be required to enable these two processive endoglucanases as efficacious exogenous fibre enzymes in pig nutrition application.

Dietary inclusion and efficient utilization of high-fiber ingredients in food animal production including pork production play integral roles in global sustainable development. This includes economic sustainability in utilizing low-cost agricultural by-products and biofuel industrial co-products^1^; societal sustainability with reduced competition for edible grains between livestock and human populations^2–4^; and environmental sustainability in reducing manure nutrient excretion and greenhouse gas emissions^2,4,7^, meanwhile striving to produce more high-quality animal protein for the world.

Efficiency of dietary fibre utilization in pigs is, in part, contributed by gut microbiota^7–9^ and is further regulated at the level of gut microbiome^10,11^. For the past several decades, dietary supplementation of various exogenous fibre enzymes that are developed through enzyme biotechnology has been widely practiced for further enhancement of dietary fibre utilization in pigs^12–16^. However, endpoint responses of in vivo efficacy are frequently inconsistent, variable and at marginal levels of improvements particularly for cellulose degradation^6,7,16–21^. Thus, further research efforts are needed to modulate gut microbiome and efficacy of exogenous fibre enzymes for improving efficiency of dietary fibre utilization in pigs.

Dietary fibre is primarily of plant cell wall origins of lignocellulosic biomass constituting of cellulose, hemicelluloses, pectin and lignin^6^. The plant cell wall originated dietary fibre has a limited surface pore size measuring at about 3.5–5.2 nm in diameter^6,22^. Currently, commercial exogenous cellulases are largely tailored from biofuel enzymes that are characterized and engineered from *Trichoderma*, *Humicola insolens* and *Aspergillus* fungal species and the *Bacillus* sp. with a large enzyme molecular size and limited penetrating property for hydrolysis of crystalline cellulosic substrates^6,23,24^. Under this context, two glycoside hydrolase family-5 (GH5) cellulases, including GH5-tCel5A1 originated and further modified from the extremely thermophilic *Thermotoga maritima* and GH5-p4818Cel5_2A screened out of the porcine hindgut microbiome, have been reported to have small molecular weights and a diameter size, thus potentially highly penetrating^6,11,25^. Both GH5-tCel5A1 and GH5-p4818Cel5_2A are processive endocellulases, are active in hydrolyzing natural crystalline cellulosic substrates and have multi-functionality towards several hemicelluloses such as β-glucans, xylan, xylogulcans, mannans, galactomannans and glucomannans^6,11,25^, thus having the potential to emerge as highly efficacious exogenous fibre biocatalysts.

Exogenous fibre enzymes are expected to be functional in the distal region of the small intestine and in the hindgut in pigs^26^ and other monogastrics in comparison with the ruminal acidic pH and microbial proteolytic environment in ruminants^27^. Exogenous fibre enzyme stability has been well recognized as major limiting factors responsible for their limited in vivo efficacy^4,7,27,28^. Thermostability and presence of intrinsic inhibitors such as phenolics in biomass have also been well documented in affecting exogenous fibre enzyme stability^4,7,29–32^. Gastrointestinal enzyme stability properties such as resistance to irreversible inactivation by gastric acidic pH along with pepsin as well as resistance to intestinal luminal residual proteases such as trypsin and chymotrypsin have also been reported in some newly characterized fiber enzymes^11,33–35^. However, less considerations have been given to carry out fibre enzyme stability research under more closely mimicked in vivo gut physiological conditions such as considering realistic residual protease activities; and food passage and retention time in most previously reported studies. Furthermore, while cysteine (Cys) residues are essential to protein folding and properties^36,37^, free thiol (−SH) groups provided by Cys residues on active sites are susceptible to oxidation, potentially leading to protein structural and functional property changes^37,38^. However, this free thiol on the active site impact is less well documented in terms of affecting exogenous fibre enzyme stability, shelf-life and in vivo efficacy. Clearly, there is a need to further investigate both GH5-tCel5A1 and GH5-p4818Cel5_2A for their stability in vitro and suitability as exogenous fibre biocatalysts in pig nutrition to warrant further biological engineering and/or potential post-fermentation enzyme processing strategies.

Under this context, it has been well documented that thermal treatment of thermophilic proteins could further induce their structure and property changes^39^, we thus wished to also investigate responses of the concerned enzyme stability properties for the heat-processed version of the thermophilic GH5-tCel5A1. We then overexpressed both GH5-tCel5A1 and GH5-p4818Cel5_2A cellulases in the endotoxin-free *E. coli* strain, referred to as the CLEARCOLIBL21 (DE3), as developed by Mamat et al.^40^. While CLEARCOLI BL21 (DE3) is readily used as an effective microbial platform for various pharmaceutical applications^40^, it is also feasible to further exploit CLEARCOLI BL21 (DE3) for our target processive cellulases’ manufacturing and governmental regulatory approval.

Therefore, the main objectives of this study were (i) to examine if predicted presence of Cys residues in catalytic sites would render GH5-tCel5A1 and GH5-p4818Cel5_2A susceptible to auto-oxidation by airborne oxygen and impair their enzyme stability in vitro; (ii) to investigate these target endocellulases’ resistance to gastric pH and pepsin under the mimicked in vitro porcine gastric conditions; and (iii) to further study these target endocellulases’ resistance to intestinal trypsin and chymotrypsin under the mimicked in vitro porcine small intestinal conditions.

## Results

### Predicting Cys residues on the target endocellulases’ catalytic sites via 3-D modelling

**Figure 1:**
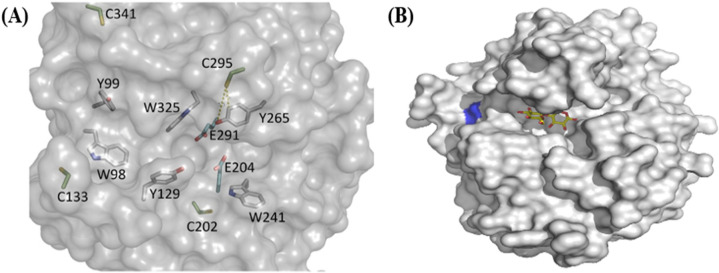
The 3-dimensional (D) modelling of the GH5-p4818Cel5_2A processive cellulase as generated by the SWISS-MODEL online server using the crystal structure of a homologous cellulase (PDB ID:1E5J) as a template. The 3-D structure of the GH5-tCel5A1 was based on PDB#: 3AOF. The 3-D structure images were generated using PYMOL (www.pymol.org). **(A)** The GH5-p4818Cel5_2A 3-D model shows four predicted cysteine (Cys) residues. C295 is likely linked to the enzyme inactivation due to auto-oxidation conditions such as when exposed to airborne O_2_ and solubilized O_2_ in aqueous buffers; and **(B)** The 3-D structure of GH5-tCel5A1 shows only one Cys residue (blue) located near the substrate binding cleft. Cellobiose as a typical end-product of this enzyme-catalyzed reaction is shown in yellow colour.

The 3-dimensional modelling (3-D) of GH5-p4818Cel5_2A is illustrated in Fig. 1A. and it has revealed four Cys residues on the enzyme’s catalytic site. One of the Cys residues labelled as C295 is located in the long tunnel-like active site and is likely associated with the enzyme inactivation as a result of exposure to auto-oxidation conditions such as airborne O_2_ and/or solubilized O_2_ in aqueous buffers. Similarly, the 3-dimensional modelling of GH5-tCel5A1 is shown in Fig. 1B and it has revealed only one Cys residue coloured in blue and is located near the substrate binding cleft of this cellulase. In addition, cellobiose, the typical end product of this GH5-tCel5A1enzyme-catalyzed reaction is shown coloured in yellow (Fig. 1B).

### These target endocellulases’ proteins were purified with SDS-PAGE analyses

**Figure 2:**
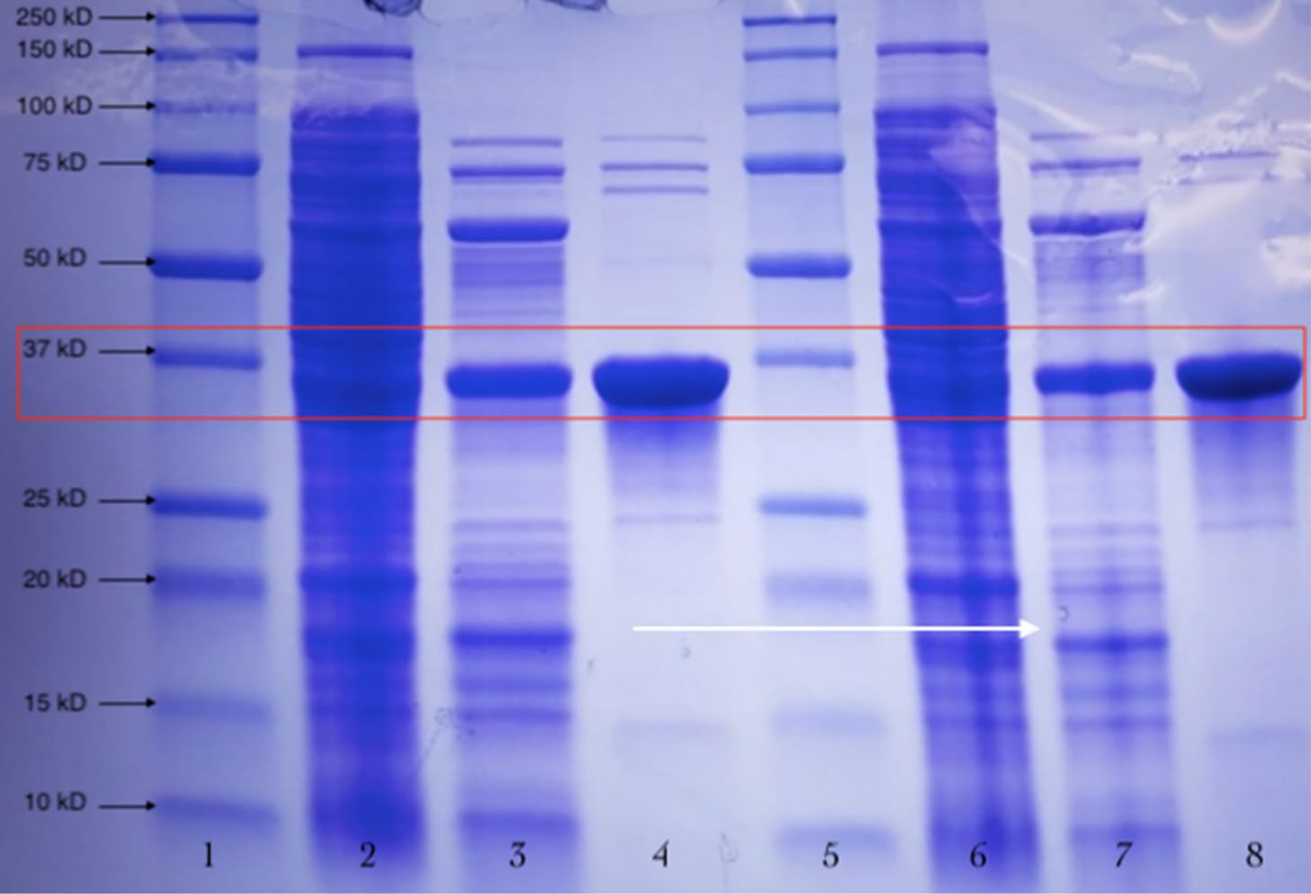
Chromatographically purified GH5-tCel5A1 cellulase, the heat-treated crude GH5-tCel5A1 cellulase extract and crude GH5-tCel5A1 cellulase extract were further analyzed by SDS-PAGE with gel stained with Coomassie blue gel and the GH5-tCel5A1 target protein at the estimated molecular weight as about 37 kDa shown in the red square. **Lanes 1** and **5**, protein molecular ladders; **Lanes 2** and **6**, crude extract of the GH5-tCel5A1; **Lanes 3** and **7**, the heat-treated GH5-tCel5A1; and **Lanes 4** and **8**, the purified GH5-tCel5A1 cellulase.

The Coomassie blue stained SDS-PAGE gel images for the GH5-tCel5A1 over-expressed enzyme samples are shown in Fig. 2. In reference to the protein molecular ladder, the purified target GH5-tCel5A1 cellulase is estimated to be about 37 kDa. Our initial enzyme assays with the purified GH5-tCel5A1 and GH5-p4818Cel5_2A shown significantly reduced enzyme activities (data not shown here) compared with the corresponding tGH5-tCel5A1 and GH5-p4818Cel5_2A over-expressed crude cell lysate samples. These discrepancies would be due to the combined exposure to the airborne O_2_ during cell lysis treatment for releasing these target enzymes and during the subsequent enzyme purification chromatography procedures, leading to the demonstrated reduction in these enzyme activities. Thus, the purified GH5-tCel5A1 and GH5-p4818Cel5_2A enzymes were not further utilized in the following in vitro stability experiments.

### Experiment 1—The target endocellulases were susceptible to auto-oxidation by airborne O_2_ in vitro

**Figure 3:**
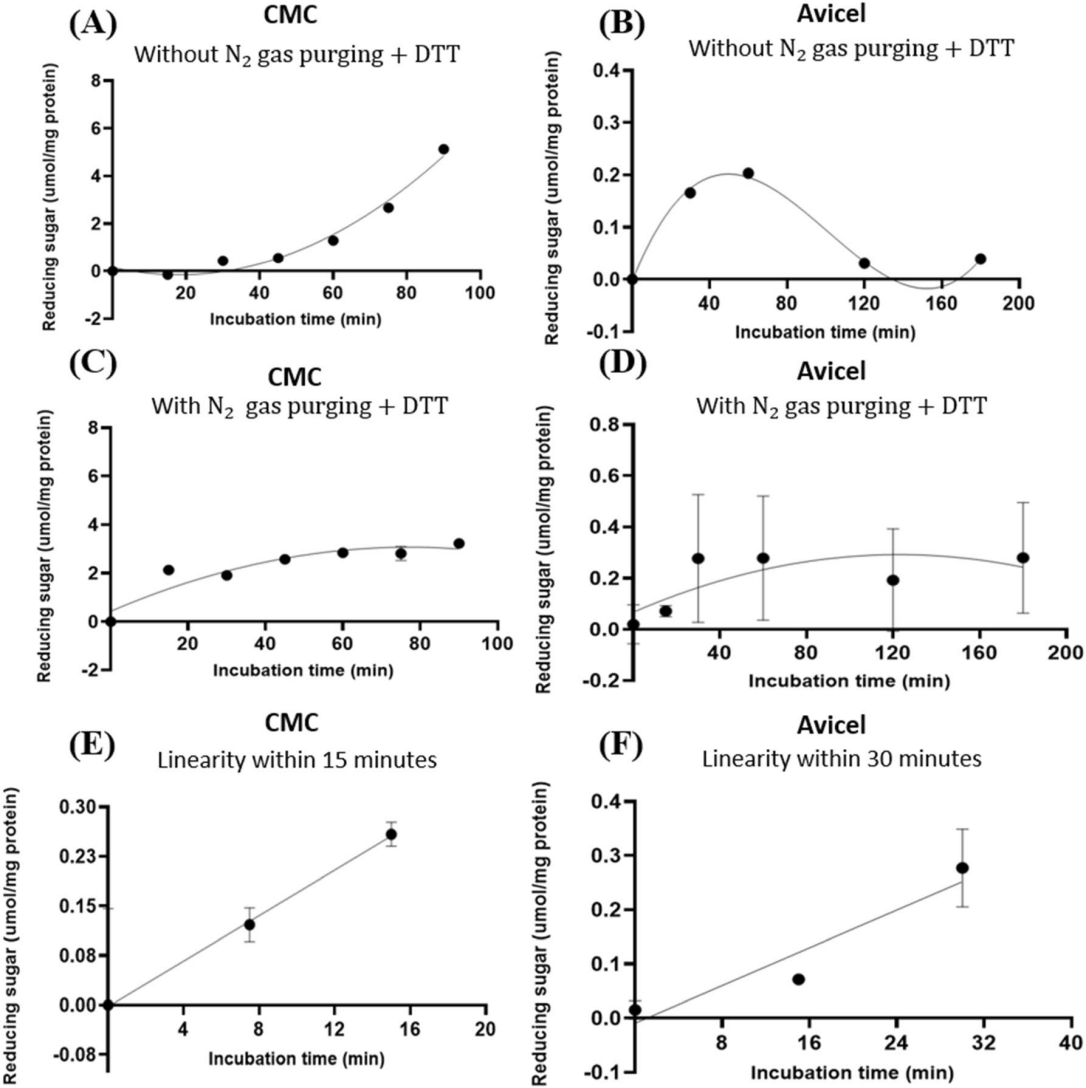
Experiment-1 of the time courses (y, mean $\pm \mathrm{SE},$ umol/mg protein) with describing model parameter estimates $\pm \mathrm{SE})$ of crude GH5-tCel5A1 enzyme preparation incubated with carboxymethyl cellulose (CMC) and Avicel as the substrate buffers in the presence or absence of N_2_ gas purging and dithiothreitol (DTT) (5 mM) for up to 90–180 min, respectively, in examining the effects of auto-oxidation on GH5-tCel5A1 hydrolysis. **(A)** Responses (y, mean $\pm \mathrm{SE}$, n = 4 at each time point) of the crude GH5-tCel5A1 enzyme preparation incubated with the CMC substrate buffer without N_2_ gas purging and the DTT (5 mM) for up to 90 min described according to a quadratic model (model parameter estimates $\pm \mathrm{SE})$ as: $Y=-0.036\left(\pm 0.013\right)x-9.0{e}^{-4}\left(\pm 1.3{e}^{-4}\right){\mathrm{x}}^{2}$, *P* = 0.472 for the intercept; *P* = 0.009 for the linear parameter estimate; and *P* < 0.0001 for the quadratic parameter estimate; ${\mathrm{R}}^{2}$ = 0.927, n = 28; **(B)** Responses (y, mean $\pm \mathrm{SE}$, n = 4 at each time point) of the crude GH5-tCel5A1 enzyme preparation incubated with the Avicel substrate buffer without N_2_ gas purging and DTT (5 mM) for up to 180 min described according to a quartic model (model parameter estimates $\pm \mathrm{SE})$ as: $Y=1.564{\left(\pm 0.481\right)\mathrm{x}}^{2}-1.041{\left(\pm 0.279\right)\mathrm{x}}^{3}+0.191{(\pm 0.049)\mathrm{x}}^{4}$, *P* = 0.244 for the intercept; *P* = 0.093 for the linear parameter estimate; *P* = 0.006 for the quadratic parameter estimate; *P* = 0.003 for the cubic parameter estimate; and *P* = 0.002 for the quartic parameter estimate;${\mathrm{R}}^{2} = 0.727,\mathrm{ n}=20$; **(C)** Responses (y, mean $\pm \mathrm{SE}$, n = 4 at each time point) of the crude GH5-tCel5A1 enzyme preparation incubated with the CMC substrate buffer with N_2_ gas purging with DTT (5 mM) for a total of 90 min established according to a quadratic model (model parameter estimates $\pm \mathrm{SE})$ as: $Y=0.474\left(\pm 0.229\right)+0.067\left(\pm 0.012\right)x-4.6{e}^{-4}(\pm 1.0{e}^{-4}){\mathrm{x}}^{2}$; *P* = 0.049 for the intercept; *P* < 0.0001 for the linear parameter estimate; and *P* = 0.002 for the quadratic parameter estimate; ${\mathrm{R}}^{2}$ = 0.837, n = 32; **(D)** Responses (y, mean $\pm \mathrm{SE}$, n = 12 at each time point) of the crude tCel5A1 enzyme preparation incubated with the Avicel substrate buffer with N_2_ gas purging and DTT (5 mM) for up to 180 min established according to a quadratic model (model parameter estimates $\pm \mathrm{SE})$ as: $Y=0.004\left(\pm 0.001\right)x-2.0{e}^{-5}(\pm 7.462{e}^{-6}){\mathrm{x}}^{2}$, *P* = 0.006 for the slope estimate and *P* = 0.038 for the quadratic parameter estimate; ${\mathrm{r}}^{2}$= 0.167, n = 72); **(E)** the linear part (y, mean $\pm \mathrm{SE}$, n = 4 at each time point) (0, 7.5 and 15 min) part of the above**-(C)** time course experiment with the crude GH5-tCel5A1 enzyme preparation incubated with the CMC buffer at 37 °C and pH 7.4 with N_2_ gas purging and DTT (5 mM) established (model parameter estimates $\pm \mathrm{SE})$ as: $y=0.149{\left(\pm 0.010\right)\mathrm{x}}$, *P* = 0.096 for the intercept and *P* < 0.0001 for the slope, ${\mathrm{r}}^{2}$ = 0.963, n = 12; and **(F)** the linear part (y, mean $\pm \mathrm{SE}$, n = 12 at each time point) (0, 15 and 30 min) of the above**-(D)** time course experiment with the crude tCel5A1 enzyme preparation incubated with the Avicel substrate buffer at 37 °C and pH 7.4 with N_2_ gas purging and DTT (5 mM) established (model parameter estimates $\pm \mathrm{SE})$ as: $y=0.008\left(\pm 0.002\right)x$, *P* = 0.909 for the intercept and *P* < 0.0001 for the slope, ${\mathrm{r}}^{2} = 0.358,\mathrm{ n}=36$.

Based on the predicted presence of one Cys residue by the preceding 3-D modelling of GH5-tCel5A1, this enzyme’s auto-oxidation susceptibility time course experiment was performed with and without ${\mathrm{N}}_{2}$ gas purging and 5 mM dithiothreitol (DTT). With using the carboxymethyl cellulose (CMC) and Avicel respectively as a soluble and an insoluble substrate, GH5-tCel5A1 displayed much delayed hydrolysis towards CMC (Fig. 3A) and a poor stability in hydrolysis of Avicel (Fig. 3B) when there was no ${\mathrm{N}}_{2}$ gas purging for depletion of O_2_ in the assay buffer, headspace (i.e., the empty space above the incubation mixture in each test tube) and absence of 5 mM DTT as an anti-oxidant. In contrast, when there was ${\mathrm{N}}_{2}$ gas purging for O_2_ depletion in the assay buffer and the headspace and the presence of 5 mM DTT, GH5-tCel5A1 exhibited a regular quadratic pattern (*P* < 0.05) hydrolysis of CMC (Fig. 3C) while maintaining a linear pattern (*P* < 0.05) of hydrolysis of Avicel (Fig. 3D). Furthermore, under the ${\mathrm{N}}_{2}$ purging and 5 mM DTT, the GH5-tCel5A1 cellulase demonstrated a linear pattern of hydrolysis (*P* < 0.05) for CMC during 0–15 min (Fig. 3E) and for Avicel during 0–30 min (Fig. 3F). These time course experimental results suggest that GH5-tCel5A1 was susceptible to auto-oxidation by airborne O_2_.

**Figure 4:**
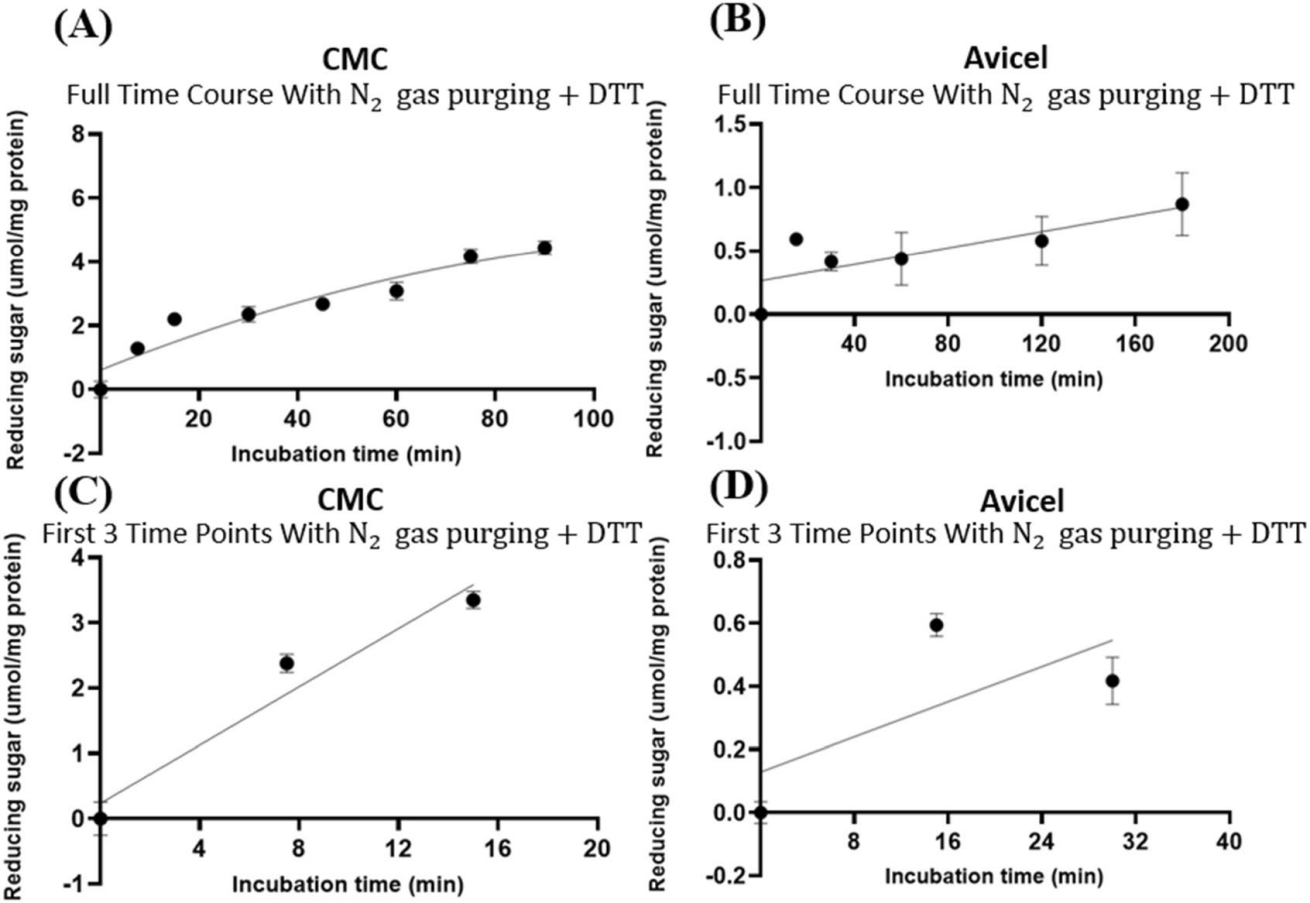
Experiment-1 of the time courses (y, mean $\pm \mathrm{SE},$ umol/mg protein) with the heat-treated crude GH5-tCel5A1 enzyme preparation incubated with the carboxymethyl cellulose (CMC) and Avicel substrate buffers at 37 °C and pH 7.4 in the presence of N_2_ gas purging and DTT at 5 mM for up to 90 and 180 min, respectively. **(A)** Responses (y, mean $\pm \mathrm{SE}$, n = 4 at each time point) of the heat-treated crude GH5-tCel5A1 enzyme preparation incubated with the CMC buffer for 90 min established according to a quadratic model (model parameter estimates $\pm \mathrm{SE})$: $Y=0.874\left(\pm 0.292\right)+0.147\left(\pm 0.017\right)x-0.001 \left(\pm 1.8{e}^{-4}\right){x}^{2},$ *P* = 0.005 for the intercept; *P* < 0.0001 for the linear and quadratic parameter estimates;${\mathrm{R}}^{2} = 0.876,\mathrm{ n}=32$; **(B)** Responses (y, mean $\pm \mathrm{SE}$, n = 12 at each time point) of the heat-treated crude GH5-tCel5A1 enzyme preparation incubated with Avicel for up to 180 min established according to a linear model (model parameter estimates $\pm \mathrm{SE})$: $y=0.242\left(\pm 0.091\right)+0.205\left(\pm 0.059\right)x$, *P* = 0.009 for the intercept; and *P* = 0.001 for the slope; ${\mathrm{r}}^{2} = 0.145,\mathrm{ n}=72$; **(C)** Linear part (y, mean $\pm \mathrm{SE}$, n = 4 at each time point) of the the first three time points (0, 7.5 and 15 min) from the above**-(A)** the time course experiment with the heat-treated crude GH5-tCel5A1 enzyme preparation incubated with the CMC buffer at 37 °C and pH 7.4 established as (model parameter estimates $\pm \mathrm{SE})$: $y=0.227{\left(\pm 0.027\right)\mathrm{x}}$, *P* = 0.511 for the intercept; and *P* < 0.0001 for the slope; ${\mathrm{r}}^{2}$ = 0.905, n = 12; and **(D)** Linear part (y, mean $\pm \mathrm{SE}$, n = 12 at each time point) of the first three time points (0, 15 and 30 min) from the above**-(B)** the time course experiment with the heat-treated crude GH5-tCel5A1 enzyme preparation incubated with the Avicel buffer at 37 °C and pH 7.4 established as (model parameter estimates $\pm \mathrm{SE})$: $y=0.015\left(\pm 0.003\right)x$, *P* = 0.105 for the intercept; and *P* < 0.0001 for the slope,${\mathrm{r}}^{2} = 0.373,\mathrm{ n}=36$.

The GH5-tCel5A1 is a hyper-thermophilic cellulase. To further characterize this cellulase, we also conducted a time course experiment with a heat-treated GH5-tCel5A1. With the presence of N_2_ purging and DTT, heat-treated GH5-tCel5A1 showed a quadratic pattern (*P* < 0.05) of hydrolysis of CMC (Fig. 4A) while maintaining a linear pattern (*P* < 0.05) of hydrolysis of Avicel (Fig. 4B). In addition, through examining results of the first three time points of the time course experiments, we established a linear pattern of hydrolysis (*P* < 0.05) for CMC during 0–15 min (Fig. 4C) and for Avicel between 0 and 30 min (Fig. 4D).

**Figure 5:**
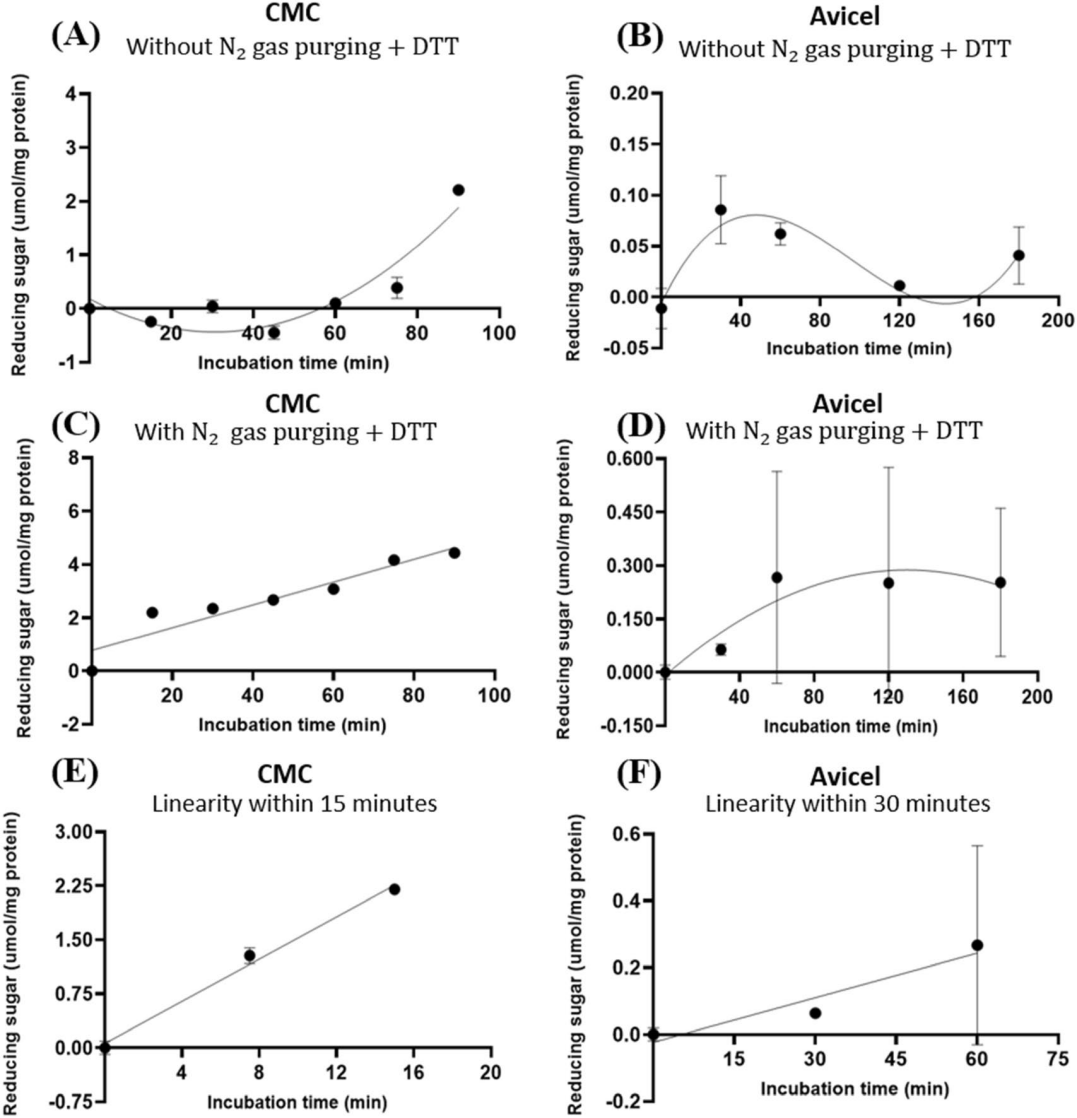
Experiment-1 of the time courses (y, mean $\pm \mathrm{SE},$ umol/mg protein) of crude GH5-p4818Cel5_2A enzyme preparation incubated with the carboxymethyl cellulose (CMC) and Avicel substrate buffers in the presence or absence of N_2_ gas purging for up to 90 and 180 min, respectively, in examining the effects of auto-oxidation on GH5-p4818Cel5_2A activity. **(A)** Responses (y, mean $\pm \mathrm{SE}$, n = 4 at each time point) of the p4818Cel5_2A enzyme preparation incubated with the CMC substrate buffer without N_2_ gas purging and DTT (5 mM) for up to 90 min established according to a quadratic model (model parameter estimates $\pm \mathrm{SE})$: $Y=-0.041\left(\pm 0.011\right)x+6.7{e}^{-4}{(\pm 1.1{e}^{-4})\mathrm{x}}^{2}$, *P* = 0.342 for the intercept; *P* = 0.001 for the linear parameter estimate; and *P* < 0.0001 for the quadratic parameter estimate; ${\mathrm{R}}^{2} = 0.777,\mathrm{ n}=28$ **(B)** Responses (y, mean $\pm \mathrm{SE}$, n = 4 at each time point) of the GH5-p4818Cel5_2A enzyme preparation incubated with the Avicel substrate buffer without N_2_ gas purging and DTT (5 mM) for up to 180 min established according to a cubic model (model parameter estimates $\pm \mathrm{SE})$ as: $Y=0.253{\left(\pm 0.059\right)\mathrm{x}}^{ }-0.193{\left(\pm 0.048\right)\mathrm{x}}^{2}+0.039{(\pm 0.010)\mathrm{x}}^{3}$, *P* = 0.064 for the intercept; *P* = 0.001 for the linear parameter estimate; *P* = 0.002 for the quadratic parameter estimate; and *P* = 0.003 for the cubic parameter estimate; ${\mathrm{R}}^{2} = 0.662,\mathrm{ n}=20$; **(C)** Responses (y, mean $\pm \mathrm{SE}$, n = 4 at each time point) of the GH5-p4818Cel5_2A enzyme preparation incubated with the CMC substrate buffer with N_2_ gas purging and DTT (5 mM) for up to 90 min established according to a linear model (model parameter estimates $\pm \mathrm{SE})$: $y=0.856\left(\pm 0.189\right)+0.042\left(\pm 0.003\right)x,$ *P* = 0.0001 for the intercept; and *P* < 0.0001 for the slope; ${\mathrm{r}}^{2}=0.874$, n = 28; **(D)** Responses (y, mean $\pm \mathrm{SE}$, n = 12 at each time point) of the GH5-p4818Cel5_2A enzyme preparation incubated with Avicel substrate buffer with N_2_ gas purging and DTT (5 mM) for up to 180 min established according to a linear model (model parameter estimates $\pm \mathrm{SE})$: $Y=0.242\left(\pm 0.91\right)+0.003\left(\pm 0.001\right)x$, *P* = 0.009 for the intercept and *P* = 0.001 for the linear parameter estimate; ${\mathrm{r}}^{2}$ = 0.145, n = 72; **(E)** Linear part (y, mean $\pm \mathrm{SE}$, n = 4 at each time point) of the (0, 7.5 and 15 min) the above**-(C)** the time course experiment with the p4818Cel5_2A enzyme preparation incubated with the CMC substrate buffer at 37 °C and pH 7.4 with N_2_ gas purging and DTT (5 mM) established as (model parameter estimates $\pm \mathrm{SE})$: $y=0.148\left(\pm 0.010\right)x$, *P* = 0.714 for the intercept; and *P* < 0.0001 for the slope $; {\mathrm{r}}^{2} = 0.965,\mathrm{ n}=12$; and **(F)** Linear part (y, mean $\pm \mathrm{SE}$, n = 12 at each time point) of the (0, 15 and 30 min) the above**-(D)** the time course experiment with the GH5-p4818Cel5_2A enzyme preparation incubated with the Avicel buffer at 37 °C and pH 7.4 with N_2_ gas purging and DTT (5 mM) established as (model parameter estimates $\pm \mathrm{SE})$: $y=0.009\left(\pm 0.002\right)x$, *P* = 0.631 for the intercept; and *P* = 0.0002 for the slope; ${\mathrm{r}}^{2} = 0.315,\mathrm{ n}=36$.

We next carried out the auto-oxidation susceptibility time course experiment with the crude GH5-p4818Cel5_2A cellulase preparation. Again, when there was no ${\mathrm{N}}_{2}$ gas purging for depletion of O_2_ in the assay buffer and the headspace and the absence of 5 mM DTT as an anti-oxidant, GH5-p4818Cel5_2A displayed much delayed hydrolysis towards CMC (Fig. 5A) and a poor stability in hydrolysis of Avicel (Fig. 5B). However, with the ${\mathrm{N}}_{2}$ gas purging treatment and presence of 5 mM DTT, GH5-p4818Cel5_2A displayed a regular quadratic pattern (*P* < 0.05) of hydrolysis for CMC (Fig. 5C) while maintaining a linear pattern (*P* < 0.05) of hydrolysis of Avicel (Fig. 5D). Additionally, under the ${\mathrm{N}}_{2}$ purging and 5 mM DTT, the GH5-p4818Cel5_2A cellulase demonstrated a linear pattern (*P* < 0.05) of hydrolysis for CMC during 0–15 min (Fig. 5E) and for Avicel between 0–30 min (Fig. 5F). These time course experimental results also indicated that GH5-p4818Cel5_2A was also vulnerable to auto-oxidation by airborne O_2_.

### Experiment 2—The target endocellulases were not very resistant to porcine gastric pH and pepsin in vitro

In order to further characterize GH5-tCel5A1, the heat-treated GH5-tCel5A1 and GH5-p4818Cel5_2A cellulases for potential in vivo pig nutrition application, the stability of these enzymes under a typical porcine gastric pH condition of 3.5 was investigated. The crude cellulase preparation samples were incubated with a gastric bicarbonate buffer at the pH 3.5 and were purged with ${\mathrm{N}}_{2}$ gas for up to 5 h and were subsequently incubated with the CMC substrate under the ${\mathrm{N}}_{2}$ gas purged condition for 15 min based upon the conditions that were established from the preceding time course and the auto-oxidation experiments.

**Figure 6:**
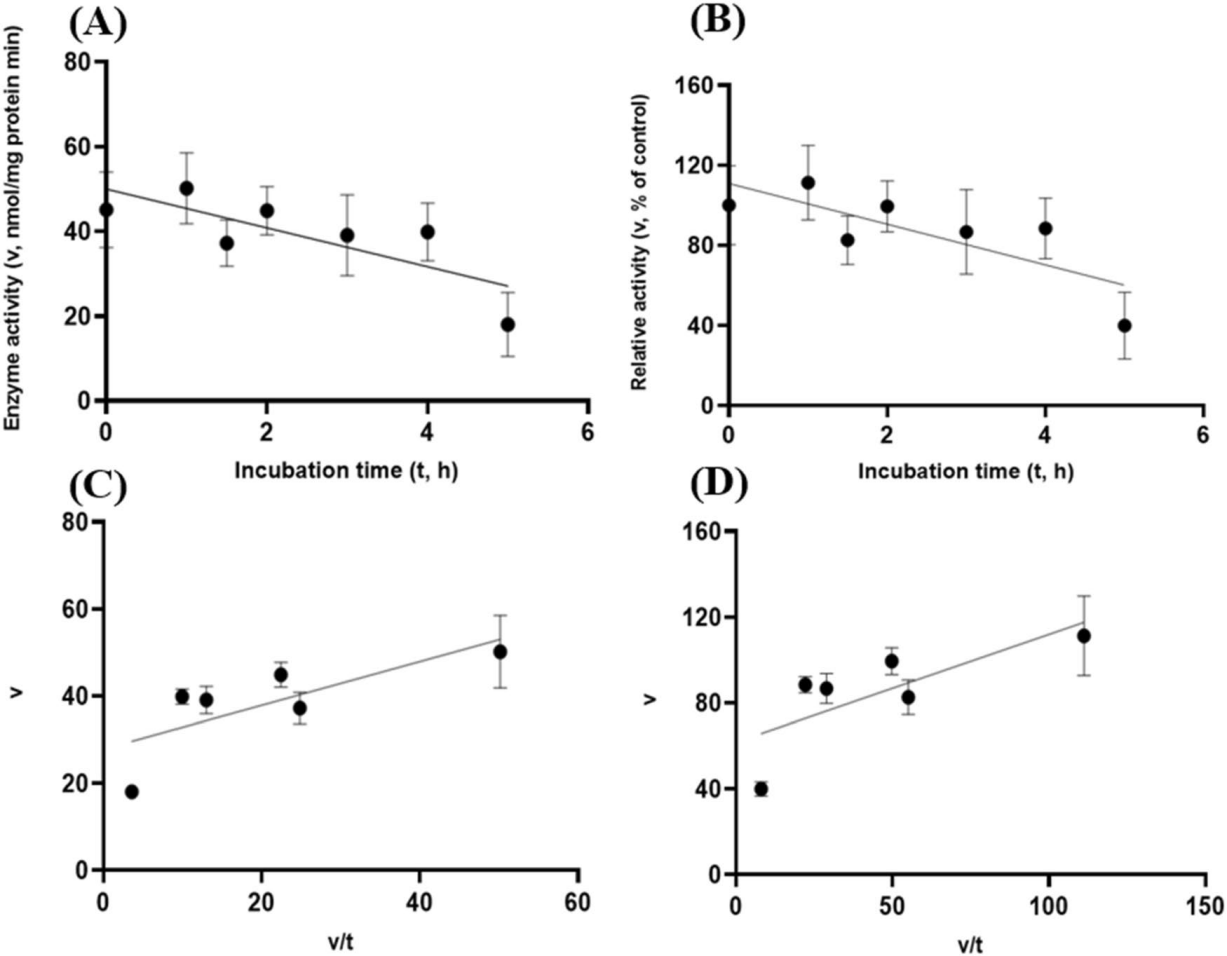
Experiment-2 of the gastric acidic pH effects (pH = 3.5) (with describing model parameter estimates $\pm \mathrm{SE})$ on GH5-tCel5A1 activity over a 5-h period after the gastric pH buffer being mixed with the crude GH5-tCel5A1 enzyme preparation prior to further incubation for measuring cellulase activity with the carboxymethyl cellulose (CMC) substrate buffer in presence of dithiothreitol (DTT) (5 mM) and N_2_ gas purging for 15 min at 37 °C. **(A)** Exponential plot of the inhibition kinetic relationship between GH5-tCel5A1 activity (v, mean $\pm \mathrm{SE},\mathrm{ n}=12\mathrm{ \,at\,each\,time\,point},$ nmol/mg protein•min) and incubation time (t, h) under gastric pH 3.5 established conditions according to the exponential response model as: $\mathrm{Y}=48.398\left(\pm 5.710\right){\mathrm{e}}^{\left[-0.099\left(\pm 0.049\right)\mathrm{x}\right)]}$, *P* < 0.0001 for the initial residual tCel5A1 activity estimate; and *P* = 0.045 for the rate constant estimate; ${\mathrm{R}}^{2}=0.080$, n = 84; and alternatively, according to the linear response model established as $\mathrm{y}=48.644\left(\pm 5.127\right)-4.193\left(\pm 1.782\right)\mathrm{x}$, *P* < 0.0001 for the intercept; and *P* = 0.021 for the slope, ${\mathrm{r}}^{2}=0.087$, n = 84; **(B)** Exponential plot of the inhibition kinetic relationship between relative GH5-tCel5A1 activity (v, mean $\pm \mathrm{SE},\mathrm{ n}=12\mathrm{ \,at\,each\,time\,point},$ % of the control group measured (100.000 ± 19.72) at 0-min exposure to the gastric pH-3.5 incubation) and incubation time (t, h) under gastric pH 3.5 conditions according to the exponential response model established as: $\mathrm{Y}=107.3\left(\pm 12.664\right){\mathrm{e}}^{\left[-0.099\left(\pm 0.049\right)\mathrm{x}\right)]}$, *P* < 0.0001 for the initial residual GH5-tCel5A1 activity estimate; and *P* = 0.045 for the rate constant estimate; ${\mathrm{R}}^{2}=0.080$, n = 84; and according to the linear response model established as $\mathrm{y}=107.9\left(\pm 11.369\right)-9.299\left(\pm 3.952\right)\mathrm{x}$, *P* < 0.0001 for all parameter estimates, ${\mathrm{r}}^{2}=0.087$, n = 84; **(C**) the Eadie-Hofstee linear plot between the GH5-tCel5A1 enzyme activity [v, mean $\pm \mathrm{SE},\mathrm{ n}=12\,\mathrm{ at\, each\, data\, point},$ nmol/mg protein•min] and the v/t ratio established as $\mathrm{y}=17.637\left(\pm 3.067\right)-1.008\left(\pm 0.113\right)\mathrm{x}$, *P* < 0.0001 for all parameter estimates; ${\mathrm{r}}^{2}=0.564$, n = 72; and **(D**) the Eadie-Hofstee linear plot between the relative GH5-tCel5A1 enzyme activity [v, mean $\pm \mathrm{SE},\mathrm{ n}=12\,\mathrm{ at\,each\,data\,point},$ % of the control group, *I*_*c*_ at 100.000 ± 19.72) measured at 0-min exposure gastric pH-3.5 incubation] and the v/t ratio established as $\mathrm{y}=39.114\left(\pm 6.801\right)-1.008\left(\pm 0.113\right)\mathrm{x}$, *P* < 0.0001 for all parameter estimates;, ${\mathrm{r}}^{2}=0.564$, n = 72.

**Figure 7:**
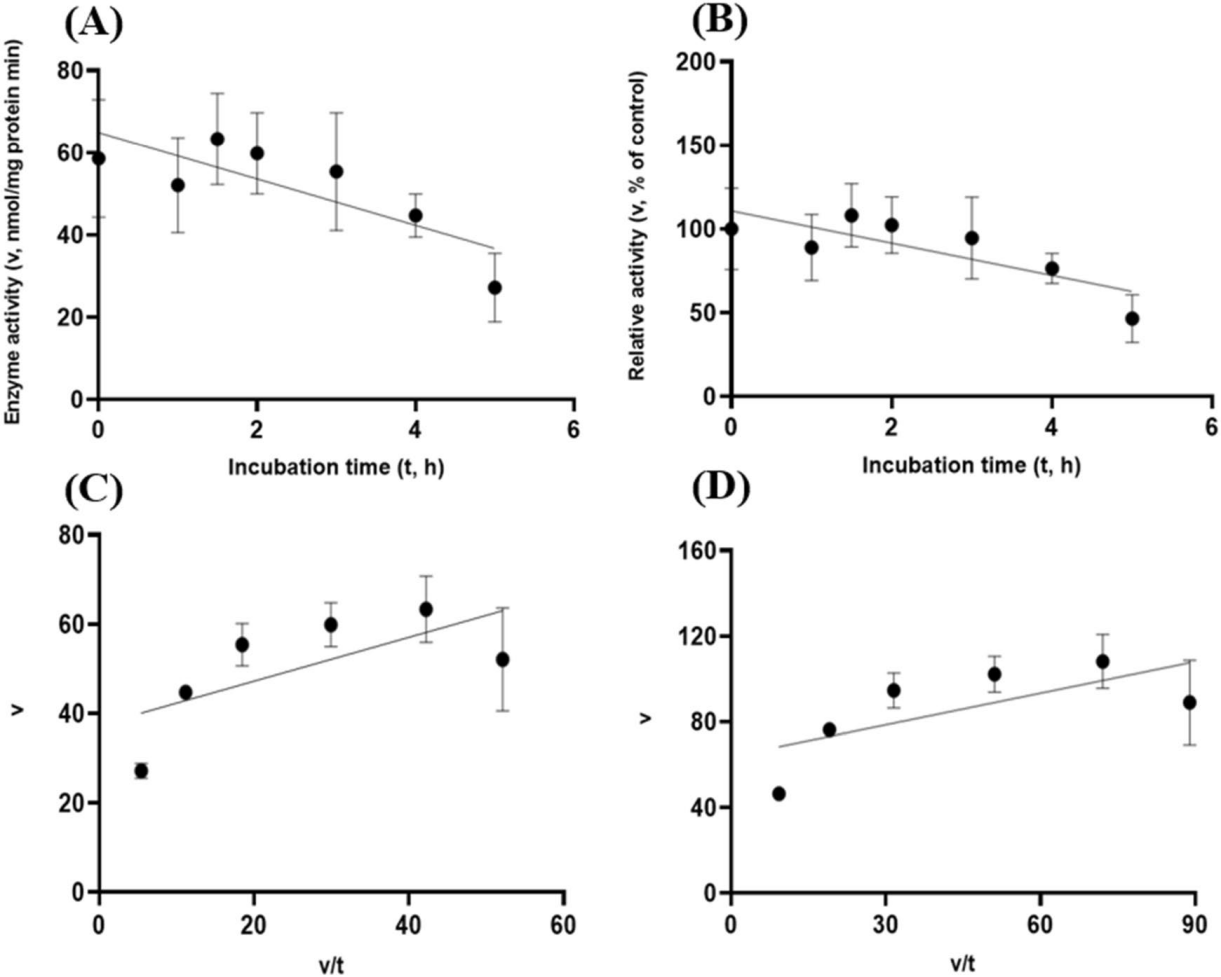
Experiment-2 of the gastric acidic pH effects (pH = 3.5) (with describing model parameter estimates $\pm \mathrm{SE})$ on the heat-treated GH5-tCel5A1 activity over a 5-h period after the gastric pH buffer being mixed with the heat-treated crude GH5-tCel5A1 enzyme preparation prior to further incubation for measuring cellulase activity with the carboxymethyl cellulose (CMC) substrate buffer in presence of dithiothreitol (DTT) (5 mM) and N_2_ gas purging for 15 min at 37 °C. **(A)** Exponential plot of the inhibition kinetic relationship between the heat-treated tCel5A1 activity [v, mean $\pm \mathrm{SE},\mathrm{ n}=12\mathrm{ \,at\,each\,time\,point},$ nmol/mg protein•min] and incubation time (t, h) under the gastric pH-3.5 conditions according to the exponential response model established as: $\mathrm{Y}=61.859\left(\pm 8.079\right){\mathrm{e}}^{(-\mathrm{x})}$, *P* < 0.0001 for the initial residual heat-treated GH5-tCel5A1 activity estimate; and *P* = 0.103 for the rate constant estimate; ${\mathrm{R}}^{2}=0.059$, n = 84; and alternatively, according to the linear response model established as $\mathrm{y}=62.513\left(\pm 7.345\right)-8.451\left(\pm 4.358\right)\mathrm{x}$, *P* < 0.0001 for the intercept; and *P* = 0.056 for the slope, ${\mathrm{r}}^{2}=0.064$, n = 84; **(B)** Exponential plot of the inhibition kinetic relationship between the relative heat-treated GH5-tCel5A1 activity (v, mean $\pm \mathrm{SE},\mathrm{ n}=12\mathrm{ \,at\,each\,time\,point},$ % of the control group measured at I_c_ of 100.000 ± 24.352) at 0-min exposure to the gastric pH 3.5 incubation) and incubation time (t, h) under gastric pH 3.5 conditions according to the exponential response model established as: $\mathrm{Y}=105.6\left(\pm 13.791\right){\mathrm{e}}^{(-\mathrm{x})}$, *P* < 0.0001 for the initial residual heat-treated GH5-tCel5A1 activity estimate; and *P* = 0.103 for the rate constant estimate, ${\mathrm{R}}^{2}=0.059$, n = 84; and alternatively, according to the linear response model established as $\mathrm{y}=106.7\left(\pm 12.537\right)-8.451\left(\pm 4.358\right)\mathrm{x}$, *P* < 0.0001 for the intercept; and *P* = 0.056 for the slope, ${\mathrm{r}}^{2}=0.087$, n = 84; **(C**) the Eadie-Hofstee linear plot between the heat treated GH5-tCel5A1 enzyme activity [v, mean $\pm \mathrm{SE},\mathrm{ n}=12\mathrm{\,at\,each\,data\,point},$ nmol/mg protein•min] and the v/t ratio established as $\mathrm{y}=20.321\left(\pm 3.744\right)+1.154\left(\pm 0.105\right)\mathrm{x}$, *P* < 0.0001 for all the parameter estimates; ${\mathrm{r}}^{2}=0.649$, n = 72; and **(D**) the Eadie-Hofstee linear plot between the relative heat-treated tCel5A1 enzyme activity (v, mean $\pm \mathrm{SE},\mathrm{ n}=12\mathrm{ \,at\,each\,data\,point},$ % of the control group measured at *I*_*c*_ of 100.000 ± 24.352 at 0-min exposure to the gastric pH 3.5 incubation) and the v/t ratio established as $\mathrm{y}=34.685\left(\pm 6.391\right)+1.153\left(\pm 0.105\right)\mathrm{x}$, *P* < 0.0001 for all parameter estimates; ${\mathrm{r}}^{2}=0.650$, n = 72.

**Figure 8:**
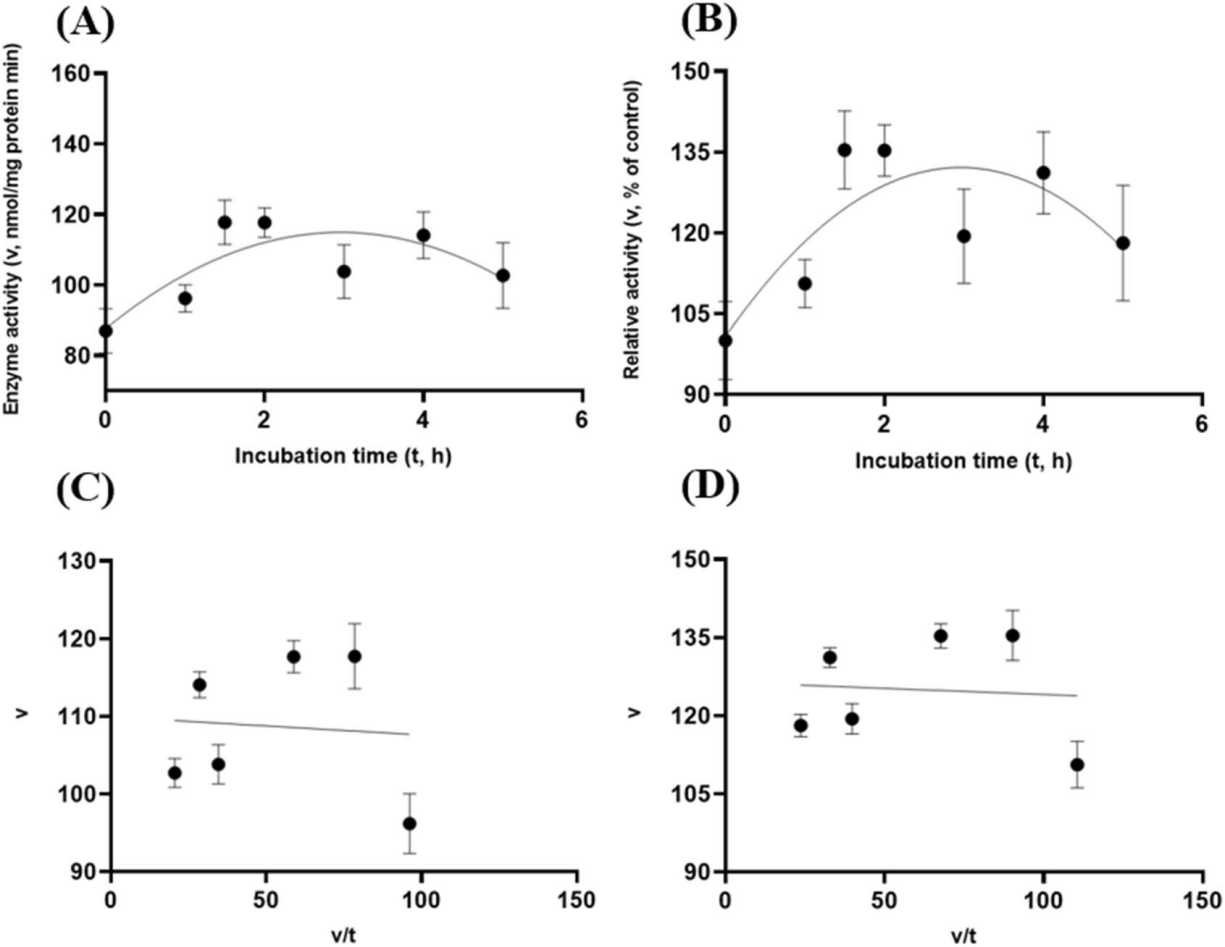
Experiment-2 of the gastric acidic pH effects (pH = 3.5) (with describing model parameter estimates $\pm \mathrm{SE})$ on GH5-p4818Cel5_2A activity over a 5-h period after the gastric pH buffer being mixed with the crude GH5-p4818Cel5_2A enzyme preparation prior to further incubation for measuring cellulase activity with the carboxymethyl cellulose (CMC) substrate buffer in presence of dithiothreitol (DTT) (5 mM) and N_2_ gas purging for 15 min at 37 °C. **(A)** Quadratic plot of the inhibition kinetic relationship between GH5-p4818Cel5_2A activity (v, mean $\pm \mathrm{SE},\mathrm{ n}=12\mathrm{ \,at\,each\,time\,point},$ nmol/mg protein•min) and incubation time (t, h) under the gastric pH 3.5 condition according to the quadratic response model established as: $\mathrm{Y}=86.308\left(\pm 6.209\right)+19.458\left(\pm 5.703\right)x-3.278(\pm 1.068){x}^{2}$, *P* < 0.0001 for intercept; *P* = 0.001 for the linear parameter estimate; and *P* = 0.003 for the quadratic parameter estimate; ${\mathrm{R}}^{2}=0.29$, n = 84; and alternatively, according to the linear response model established as $\mathrm{y}=99.305\left(\pm 4.771\right)+2.638\left(\pm 1.658\right)\mathrm{x}$, *P* < 0.0001 for the intercept; and *P* = 0.116 for the slope; ${\mathrm{r}}^{2}=0.207$, n = 84; **(B)** Quadratic plot of the inhibition kinetic relationship between the relative GH5-p4818Cel5_2A activity (v, mean $\pm \mathrm{SE},\mathrm{ n}=12\mathrm{ \,at\,each\,time\,point},$ % of the control group measured at I_C_ of 100.000 ± 7.523 at 0-min exposure to the gastric pH 3.5 incubation) and incubation time (t, h) under the gastric pH-3.5 condition according to the quadratic response model established as: $\mathrm{Y}=99.245\left(\pm 7.139\right)+22.374\left(\pm 6.558\right)x-3.769(\pm 1.228){x}^{2}$, *P* < 0.0001 for intercept; *P* = 0.001 for the linear parameter estimate; and *P* = 0.003 for the quadratic parameter estimate; ${\mathrm{R}}^{2}=0.29$, n = 84; and alternatively, according to the linear response model established as $\mathrm{y}=114.2\left(\pm 5.486\right)$, *P* < 0.0001 for the intercept; and *P* = 0.116 for the slope, ${\mathrm{r}}^{2}=0.207$, n = 84; **(C**) the Eadie-Hofstee linear plot between the GH5-p4818Cel5_2A enzyme activity (v, mean $\pm \mathrm{SE},\mathrm{ n}=12\mathrm{ \,at\,each\,data\,point},$ nmol/mg protein•min) and the v/t ratio established as $\mathrm{y}=98.265\left(\pm 5.708\right)+0.200\left(\pm 0.096\right)\mathrm{x}$, *P* < 0.0001; for the intercept and *P* = 0.041 for the slope, ${\mathrm{r}}^{2}=0.059$, n = 72; and **(D**) the Eadie-Hofstee linear plot between the relative GH5-p4818Cel5_2A enzyme activity (v, mean $\pm \mathrm{SE},\mathrm{ n}=12\mathrm{ \,at\,each\,data\,point},$ % of the control group measured at *I*_*C*_ of 100.000 ± 7.523) at 0-min exposure to the gastric pH 3.5 incubation) and the v/t ratio established as $\mathrm{y}=113.000\left(\pm 6.563\right)+0.200\left(\pm 0.096\right)\mathrm{x}$, *P* < 0.0001 for the intercept; and *P* = 0.041 for the slope, ${\mathrm{r}}^{2}=0.05$, n = 72.

Albeit of very low coefficients of determination (r^2^ and R^2^) observed, there were both exponential decay curve and linear relationships (*P* < 0.05) between the enzyme activity and incubation time under the gastric acidic pH (pH = 3.5) condition for both GH5-tCel5A1 and the heat-treated GH5-tCel5A1 crude enzyme preparations as shown in Figs. 6A–B and 7A–B, respectively. However, there was only a quadratic curve response (*P* < 0.05) between the enzyme activity and incubation time observed under the gastric acidic pH (pH = 3.5) condition for GH5-p4818Cel5_2A (Fig. 8A–B). These gastric acidic pH (pH = 3.5) enzyme inhibition kinetics over the 5-h period were further examined utilizing the Eadie-Hofstee linear regression analyses and were visualized in Fig. 6C–D for GH5-tCel5A1; Fig. 7C–D for the heat-treated tCel5A1; and Fig. 8C–D for GH5-p4818Cel5_2A, respectively.

These gastric acidic pH (pH = 3.5) enzyme inhibition kinetics are further summarized in Table 1 for all of the three target enzymes. With the *IC*_*50*_ = 1.01 and 1.05 h respectively observed for both GH5-tCel5A1 and the heat-treated GH5-tCel5A1 cellulases, their corresponding relative maximal enzyme activity inhibition (i.e., ${I}_{max}$) was estimated to be at 60.88 vs. 65.31%, clearly showing differences in this key inhibition kinetic endpoint between GH5-tCel5A1 and its heat-treated version of the GH5-tCel5A1 cellulase. In contrast, GH5-p4818Cel5_2A had an estimated maximal enzyme activity inhibition ${I}_{max}$ = 0. These gastric acidic pH (pH = 3.5) enzyme inhibition experiments demonstrated that although the GH5-p4818Cel5_2A activity was not affected by the gastric acidic pH, the GH5-tCel5A1 activity was sensitive to the gastric acidic pH and could lose more than 60% of its activity. The heat-treatment of GH5-tCel5A1 cellulase was shown ineffective to improve GH5-tCel5A1 stability to the gastric acidic pH.

**Table 1:** Comparative summary of the Experiment-2 in vitro inhibition kinetics^1^ of GH5-tCel5A1, the heat-treated GH5-tCel5A1 and GH5-p4818Cel5_2A under the gastric acidic pH of 3.5 obtained through the Eadie-Hofstee linear regression analyses.

Item	Inhibition kinetic parameter estimates				
	${I}_{max}$ ^2^		${I}_{min}$ ^3^	${I}_{c}$ ^4^	${IC}_{50}$ ^5^
*Cellulase activity (nmol/mg protein·min)*					
GH5-tCel5A1	27.45 $\pm 8.89$		17.63 $\pm 3.07$	45.09 $\pm 8.89$	$1.01\pm 0.11$
Heated GH5-tCel5A1	38.27 $\pm 14.27$		20.32 $\pm 3.74$	58.59 $\pm 14.27$	1.05 $\pm 0.12$
GH5-p4818Cel5_2A	${-11.30\pm 6.31}^{7}$		98.27 $\pm 5.71$	$86.96\pm 6.31$	0.83 $\pm 0.07$
*Relative cellulase activity (% of the control)*^6^					
GH5-tCel5A1	60.89 $\pm 19.72$		39.11 $\pm 6.80$	100.00 $\pm 19.72$	$1.01\pm 0.11$
Heated GH5-tCel5A1	65.3 $1\pm 24.35$		34.69 $\pm 6.39$	100.00 $\pm 24.35$	1.05 $\pm 0.12$
GH5-p4818Cel5_2A	${-13.00\pm 7.25}^{7}$		$113.00\pm 6.56$	100.00 $\pm 7.25$	0.83 $\pm 0.07$

^1^Values are parameter estimates or means $\pm$ SE (n = 72) obtained through the Eadie-Hofstee regression linear analyses, representing the inhibition kinetic parameter estimates obtained from the three gastric acidic pH 3.5 inhibition experiments with 4 replicates per time point in each experiment, as shown in Figs. 6, 7 and 8.

^2^ ${I}_{max}$ is the maximal magnitude of inhibition in the cellulase activity (nmol/mg protein·min or % of the control, *I*_*c*_; means $\pm$ SE, n = 12) calculated according to Eq. (2).

^3^ ${I}_{min}$ is the minimal residual cellulase activity (nmol/mg protein·min or % of the control, *I*_*c*_; parameter estimate $\pm$ SE, n = 72) under the acidic pH 3.5 inhibition condition obtained from Eq. (1).

^4^ ${I}_{c}$ is the mean cellulase activity of the control group (nmol/mg protein·min or % of the control, means $\pm$ SE, n = 12).

^5^ ${IC}_{50}$ is the incubation time (h, parameter estimate $\pm$ SE, n = 72) required to reach the half maximal inhibition of the cellulase activity.

^6^Relative cellulase activity (%, means $\pm$ SE, n = 12) calculated as percentage of the control group (*I*_*c*_) measured at 0-min exposure to the gastric pH-3.5 incubation.

^7^ ${I}_{max}$ value was non-significant from 0 (*P* > 0.05) as compared by the pooled t-test.

**Figure 9:**
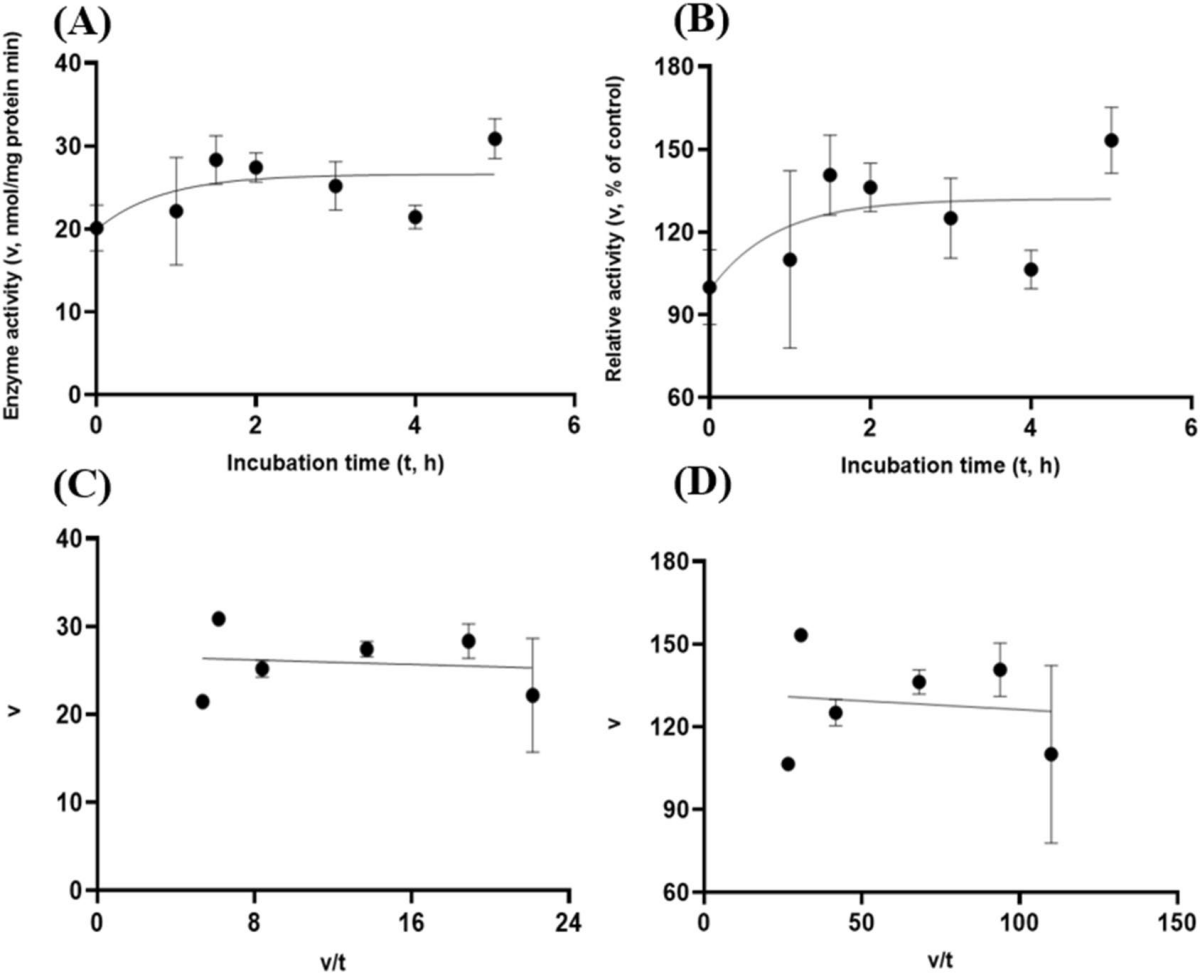
Experiment-2 of the gastric acidic pH and pepsin (274 U/mL) effects (with describing model parameter estimates $\pm \mathrm{SE})$ on GH5-tCel5A1 activity over a 5-h period after the gastric pH and pepsin buffer being mixed with the crude GH5-tCel5A1 enzyme preparation for incubation at the resulting mixture pH = 3.5 and pepsin at 274 U/mL prior to further incubation for measuring cellulase activity with the carboxymethyl cellulose (CMC) substrate buffer in presence of dithiothreitol (DTT) (5 mM) and N_2_ gas purging for 15 min at 37 °C. **(A)** Exponential plot of the inhibition kinetic relationship between the GH5-tCel5A1 activity (v, mean $\pm \mathrm{SE},\mathrm{ n}=12\mathrm{ \,at\,each\,time\,point},$ nmol/mg protein•min) and incubation time (t, h) under the gastric pH = 3.5 and pepsin (274 U/mL) conditions established according to the exponential response model as: $\mathrm{Y}=23.156\left(\pm 2.157\right){\mathrm{e}}^{(-\mathrm{x})}$, *P* < 0.0001 for the initial residual tCel5A1 activity estimate; and *P* = 0.228 for the rate constant estimate; ${\mathrm{R}}^{2}=0.072$, n = 84; and alternatively, according to the linear response model established as $\mathrm{y}=23.084\left(\pm 2.254\right)+0.949\left(\pm 0.783\right)\mathrm{x}$, *P* < 0.0001 for the intercept; and *P* = 0.229 for the slope, ${\mathrm{r}}^{2}=0.072$, n = 84; **(B)** Exponential plot of the inhibition kinetic relationship between the relative GH5-tCel5A1 activity (v, mean $\pm \mathrm{SE},\mathrm{ n}=12\mathrm{ \,at\,each\,time\,point},$ % of the control group measured (100.000 ± 19.716) at the 0-min exposure to the gastric pH-3.5 and pepsin-274 U/mL incubation) and incubation time (t, h) under the gastric pH 3.5 and pepsin (274 U/mL) conditions according to the exponential response model established as: $\mathrm{Y}=115.000\left(\pm 10.715\right){\mathrm{e}}^{(\mathrm{x})}$, *P* < 0.0001 for the initial residual GH5-tCel5A1 activity estimate; and *P* = 0.228 for the rate constant estimate, ${\mathrm{R}}^{2}=0.07$, n = 84; and alternatively, according to the linear response model established as $\mathrm{y}=114.600\left(\pm 11.193\right)+4.711\left(\pm 3.891\right)x$, *P* < 0.0001 for the intercept; and *P* = 0.229 for the slope, ${\mathrm{r}}^{2}=0.072$, n = 84; **(C**) the Eadie-Hofstee linear plot between the tCel5A1 enzyme activity (v, mean $\pm \mathrm{SE},\mathrm{ n}=12\mathrm{ \,at\,each\,data\,point},$ nmol/mg protein•min) and the v/t ratio established as $\mathrm{y}=17.432\left(\pm 1.455\right)+0.703\left(\pm 0.086\right)\mathrm{x}$, *P* < 0.0001 for all parameter estimates, ${\mathrm{r}}^{2}=0.551$, n = 72; and **(D**) the Eadie-Hofstee linear plot between the relative GH5-tCel5A1 enzyme activity (v, mean $\pm \mathrm{SE},\mathrm{ n}=12\mathrm{ \,at\,each\,data\,point},$ % of the control group measured (100.000 ± 19.716) at the 0-min exposure to the gastric pH-3.5 and pepsin-274 U/mL incubation) and the v/t ratio established as $\mathrm{y}=86.577\left(\pm 7.228\right)+0.703\left(\pm 0.086\right)\mathrm{x}$, *P* < 0.0001 for all parameter estimates; ${\mathrm{r}}^{2}=0.551$, n = 72.

**Figure 10:**
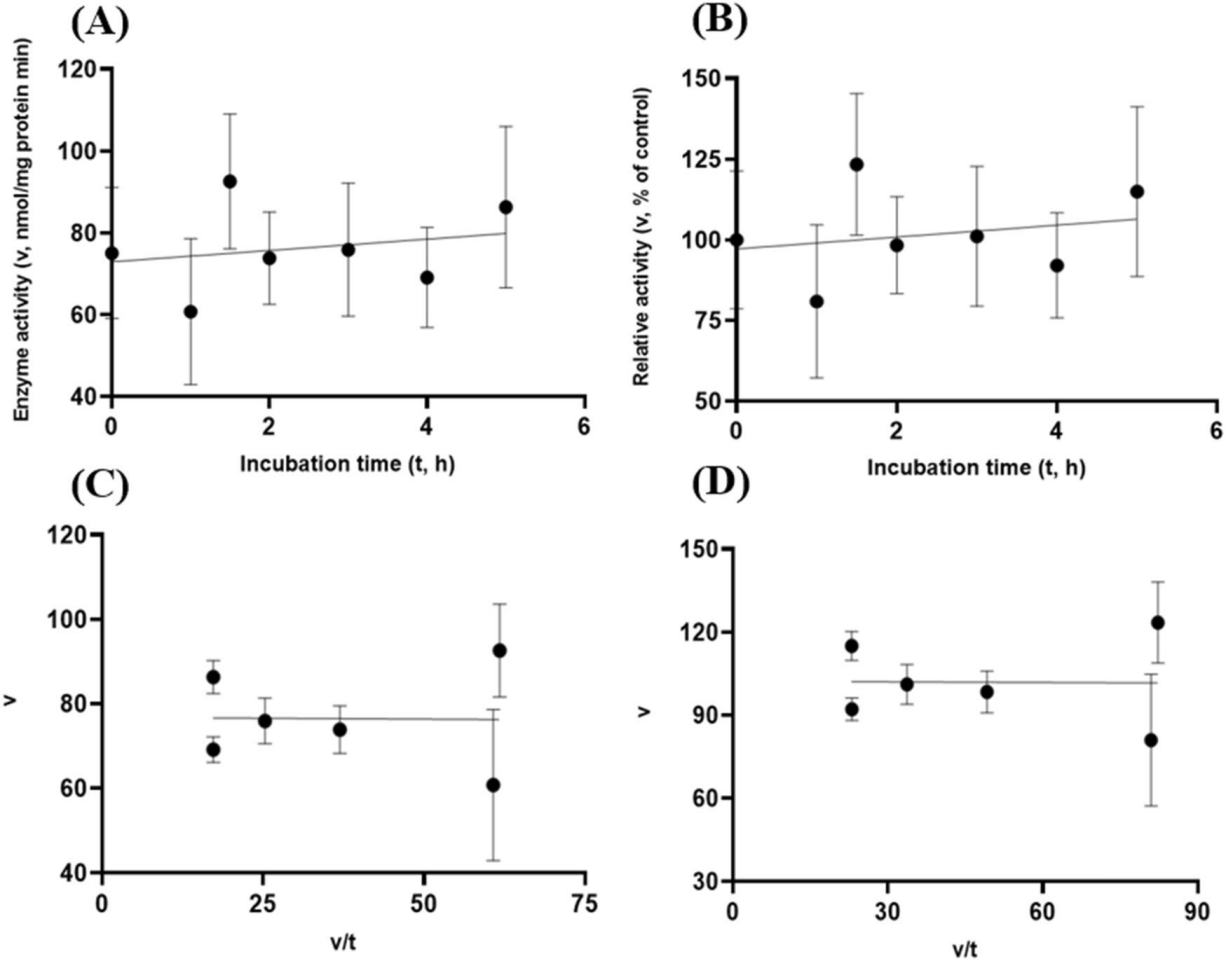
Experiment-2 of the gastric acidic pH and pepsin (274 U/mL) effects (with describing model parameter estimates $\pm \mathrm{SE})$ on heat-treated GH5-tCel5A1 activity over a 5-h period after the gastric pH and pepsin buffer being mixed with the heat-treated crude GH5-tCel5A1 enzyme preparation for incubation at the resulting mixture pH = 3.5 and pepsin at 274 U/mL prior to further incubation for measuring cellulase activity with the carboxymethyl cellulose (CMC) substrate buffer in presence of dithiothreitol (DTT) (5 mM) and N_2_ gas purging for 15 min at 37 °C. **(A)** Exponential plot of the inhibition kinetic relationship between the heat-treated GH5-tCel5A1 activity (v, mean $\pm \mathrm{SE},\mathrm{ n}=12\mathrm{ \,at\,each\,time\,point},$ nmol/mg protein•min) and incubation time (t, h) under the gastric pH 3.5 and pepsin (274 U/mL) conditions established according to the exponential response model as: $\mathrm{Y}=73.926\left(\pm 10.537\right){\mathrm{e}}^{(-\mathrm{x})}$, *P* < 0.0001 for the initial residual heat-treated tCel5A1 activity estimate; and *P* = 0.764 for the rate constant estimate; ${\mathrm{R}}^{2}=0.024$, n = 84; and alternatively, established according to the linear response model as $\mathrm{y}=73.947\left(\pm 10.717\right)+1.100\left(\pm 3.725\right)x$, *P* < 0.0001 for the intercept; and *P* = 0.769 for the slope, ${\mathrm{r}}^{2}=0.024$, n = 84; **(B)** Exponential plot of the inhibition kinetic relationship between the relative heat treated GH5-tCel5A1 activity (v, mean $\pm \mathrm{SE},\mathrm{ n}=12\mathrm{ \,at\,each\,time\,point},$ % of the control group measured at I_C_ of 100.000 ± 21.379 at the 0-min exposure to the gastric pH-3.5 and pepsin-274 U/mL incubation) and incubation time under gastric pH 3.5 and pepsin (274 U/mL) conditions according to the exponential response model established as: $\mathrm{Y}=104.600\left(\pm 14.337\right){\mathrm{e}}^{(-\mathrm{x})}$, *P* < 0.0001 for the initial residual heat treated GH5-tCel5A1 activity estimate; and *P* = 0.994 for the rate constant estimate, ${\mathrm{R}}^{2}=0.026$, n = 84; and according to the linear response model established as $\mathrm{y}=104.600\left(\pm 14.343\right)+0.0391\left(\pm 4.932\right)x$, *P* < 0.0001 for the intercept and *P* = 0.994 for the slope, ${\mathrm{r}}^{2}=0.026$, n = 84; **(C**) the Eadie-Hofstee linear plot between the heat-treated tCel5A1 enzyme activity (v, mean $\pm \mathrm{SE},\mathrm{ n}=12\mathrm{ \,at\,each\,data\,point},$ nmol/mg protein•min) and the v/t ratio established as $\mathrm{y}=38.483\left(\pm 6.445\right)+1.053\left(\pm 0.124\right)\mathrm{x}$, *P* < 0.0001 for all parameter estimates; ${\mathrm{r}}^{2}=0.524$, n = 72; and **(D**) the Eadie-Hofstee linear plot between the relative heat-treated GH5-tCel5A1 enzyme activity (v, mean $\pm \mathrm{SE},\mathrm{ n}=12\mathrm{ \,at\,each\,data\,point},$ % of the control group measured at *I*_*C*_ of 100.000 ± 21.379) at the 0-min exposure to the gastric pH-3.5 and pepsin-274 U/mL incubation) and the v/t ratio established as $\mathrm{y}=51.280\left(\pm 8.588\right)+1.053\left(\pm 0.124\right)\mathrm{x}$, *P* < 0.0001 for all parameter estimates; ${\mathrm{r}}^{2}=0.524$, n = 72.

**Figure 11:**
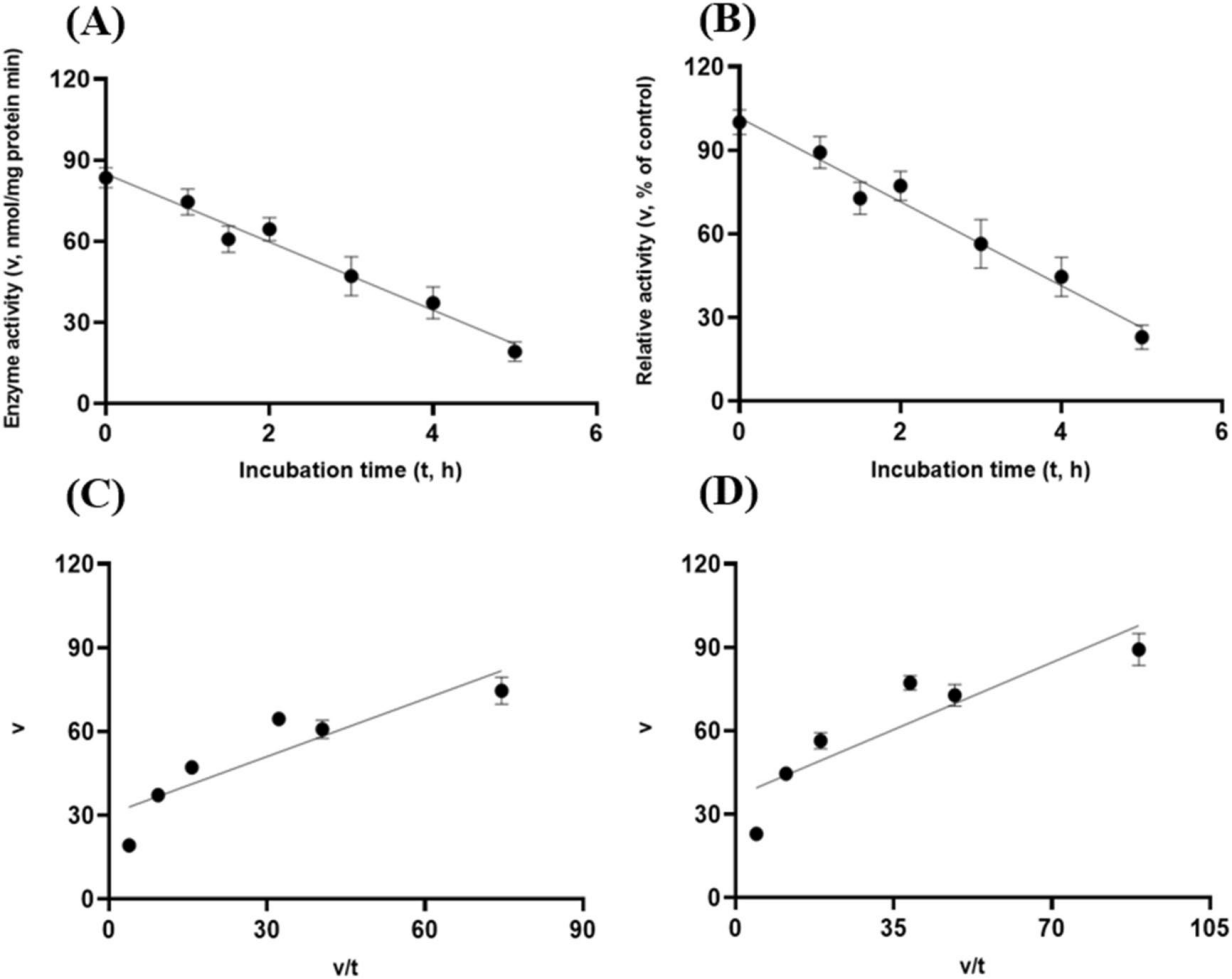
Experiment-2 of the gastric acidic pH and pepsin (274 U/mL) effects (with describing model parameter estimates $\pm \mathrm{SE})$ on GH5-p4818Cel5_2A activity over a 5-h period after the gastric pH and pepsin buffer being mixed with the GH5-p4818Cel5_2A enzyme preparation for incubation at the resulting mixture pH = 3.5 and pepsin at 274 U/mL prior to further incubation for measuring cellulase activity with the carboxymethyl cellulose (CMC) substrate buffer in presence of dithiothreitol (DTT) (5 mM) and N_2_ gas purging for 15 min at 37 °C. **(A)** Linear plot of the inhibition kinetic relationship between the GH5-p4818Cel5_2A activity (v, mean $\pm \mathrm{SE},\mathrm{ n}=12\mathrm{ \,at\,each\,time\,point},$ nmol/mg protein•min) and incubation time (t, h) under the gastric pH 3.5 and pepsin (274 U/mL) conditions established according to the linear response model as: $\mathrm{y}=85.348\left(\pm 3.415\right)-12.702\left(\pm 1.187\right)\mathrm{x}$, *P* < 0.0001 for all parameter estimates; ${\mathrm{r}}^{2}=0.606$, n = 84; and alternatively, according to the exponential response model established as $\mathrm{Y}=89.186\left(\pm 4.359\right){\mathrm{e}}^{[-0.227(\pm 0.026)\mathrm{x}]}$, *P* < 0.0001 all parameter estimates; ${\mathrm{R}}^{2}=0.581$, n = 84; **(B)** Linear plot of the inhibition kinetic relationship between the relationship of relative GH5-p4818Cel5_2A activity (v, mean $\pm \mathrm{SE},\mathrm{ n}=12\mathrm{ \,at\,each\,time\,point},$ % of the control group measured at I_C_ of 100.000 ± 4.416) at the 0-min exposure to the gastric pH-3.5 and pepsin-274 U/mL incubation) and incubation time under gastric pH 3.5 and pepsin (274 U/mL) conditions according to the linear response model established as: $\mathrm{y}=102.1\left(\pm 4.088\right)-15.202\left(\pm 1.421\right)\mathrm{x}$, *P* < 0.0001 for all parameter estimates, ${\mathrm{r}}^{2}=0.606$, n = 84; and established according to the exponential response model as $\mathrm{Y}=106.7\left(\pm 5.218\right){\mathrm{e}}^{[-0.227(\pm 0.026)\mathrm{x}]}$, *P* < 0.0001 all parameter estimates, ${\mathrm{R}}^{2}=0.581$, n = 84; **(C**) the Eadie-Hofstee linear plot between the GH5-p4818Cel5_2A enzyme activity (v, mean $\pm \mathrm{SE},\mathrm{ n}=12\mathrm{ \,at\,each\,data\,point},$ nmol/mg protein•min) and the v/t ratio established as $\mathrm{y}=26.418\left(\pm 2.663\right)+0.829\left(\pm 0.069\right)\mathrm{x}$, *P* < 0.0001 for all parameter estimates, ${\mathrm{r}}^{2}=0.677$, n = 72; and **(D**) the Eadie-Hofstee linear plot between the relative GH5-p4818Cel5_2A enzyme activity (v, mean $\pm \mathrm{SE},\mathrm{ n}=12\mathrm{ \,at\,each\,data\,point},$ % of the control group measured at *I*_*C*_ of 100.000 ± 4.416) at the 0-min exposure to the gastric pH-3.5 and pepsin-274 U/mL incubation) and the v/t ratio established as $\mathrm{y}=31.618\left(\pm 3.187\right)+0.829\left(\pm 0.070\right)\mathrm{x}$, *P* < 0.0001 for all parameter estimates, ${\mathrm{r}}^{2}=0.677$, n = 72.

To further characterize tCel5A1, the heat-treated GH5-tCel5A1 and GH5-p4818Cel5_2A cellulases for their overall in vitro porcine gastric stability, the crude cellulase preparation samples were incubated at the pH 3.5 with pepsin (274 U/mL) and the ${\mathrm{N}}_{2}$ gas purging treatment. However, both exponential decay curve and linear relationships were not observed (*P* > 0.05) between the enzyme activity and incubation time under the gastric acidic pH (pH = 3.5) with pepsin (274 U/mL) condition for both GH5-tCel5A1 and the heat-treated GH5-tCel5A1 crude enzyme preparations shown in Figs. 9A–B and 10A–B, respectively. Whereas there were exponential decay curve and linear relationships (*P* < 0.05) between the enzyme activity and incubation time observed under the gastric acidic pH (pH = 3.5) with pepsin (274 U/mL) condition for GH5-p4818Cel5_2A (Fig. 11A–B).

These gastric acidic pH (pH = 3.5) with pepsin (274 U/mL) enzyme inhibition kinetics over the 5-h period were further examined through utilizing the Eadie-Hofstee linear regression analyses and were visualized in Fig. 9C–D for tCel5A1; Fig. 10C–D for the heat-treated GH5-tCel5A1; and Fig. 11C–D for GH5-p4818Cel5_2A, respectively. These gastric acidic pH (pH = 3.5) with pepsin (274 U/mL) enzyme inhibition kinetics are further summarized in Table 2 for all of the three target enzymes. With the *IC*_*50*_ = 0.70 and 1.05 h respectively observed for both GH5-tCel5A1 and the heat-treated GH5-tCel5A1 cellulases, their corresponding relative maximal enzyme activity inhibition (i.e., ${I}_{max}$) was estimated to be at 13.42 vs. 48.72%, clearly illustrating differences in these key inhibition kinetic endpoints between GH5-tCel5A1 and its heat-treated version of the GH5-tCel5A1 cellulase by the gastric pepsin (274 U/mL) at the acidic pH (pH = 3.5). Furthermore, GH5-p4818Cel5_2A had an estimated relative maximal enzyme activity inhibition ${I}_{max}$ = 68.38% with the *IC*_*50*_ = 0.83 h by the gastric pepsin (274 U/mL) at the acidic pH (pH = 3.5).

**Table 2:** Comparative summary of the Experiment-2 in vitro inhibition kinetics^1^ of GH5-tCel5A1, the heat-treated GH5-tCel5A1 and GH5-p4818Cel5_2A under the gastric acidic pH of 3.5 and pepsin (274 U/mL) obtained through the Eadie-Hofstee linear regression analyses.

*Item*	Inhibition kinetic parameter estimates				
	${I}_{max}$ ^*2*^		${I}_{min}$ ^*3*^	${I}_{c}$ ^*4*^	${IC}_{50}$ ^*5*^
**Cellulase activity (nmol/mg protein·min)**					
*GH5-tCel5A1*	*2.70* $\pm 3.78$		*17.43* $\pm 1.46$	*20.13* $\pm 3.78$	*0.70* $\pm 0.09$
*Heated GH5-tCel5A1*	*36.56* $\pm 16.04$		*38.48* $\pm 6.44$	*74.05* $\pm 16.04$	*1.05* $\pm 0.12$
*GH5-p4818Cel5_2A*	*57.14* $\pm 3.69$		*26.42* $\pm 2.66$	*83.55* $\pm 3.69$	*0.83* $\pm 0.07$
**Relative cellulase activity (% -20of the control)**^*6*^					
*GH5-tCel5A1*	*13.42* $\pm 19.72$		*86.58* $\pm 7.23$	*100.00* $\pm 19.72$	*0.70* $\pm 0.09$
*Heated GH5-tCel5A1*	*48.72* $\pm 21.38$		*51.28* $\pm 8.59$	*100.00* $\pm 21.38$	*1.05* $\pm 0.12$
*GH5-p4818Cel5_2A*	*68.38* $\pm 4.42$		*31.62* $\pm 3.19$	*100.00* $\pm 4.42$	*0.83* $\pm 0.07$

^1^Values are parameter estimates or means $\pm$ SE (n = 72) obtained through the Eadie-Hofstee regression linear analyses, representing the inhibition kinetic parameter estimates obtained from the three gastric acidic pH 3.5 and pepsin (274 U/mL) inhibition experiments with 4 replicates per time point in each experiment, as shown in Figs. 9, 10, 11.

^2^ ${I}_{max}$ is the maximal magnitude of inhibition in the cellulase activity (nmol/mg protein·min or % of the control, *I*_*c*_*;* means $\pm$ SE, n = 12) calculated according to Eq. (2).

^3^ ${I}_{min}$ is the minimal residual cellulase activity (nmol/mg protein·min or % of the control *I*_*c*_; parameter estimate $\pm$ SE, n = 72) under the combined gastric acidic pH 3.5 and pepsin (274 U/mL) inhibition conditions obtained from Eq. (1).

^4^ ${I}_{c}$ is the mean cellulase activity of the control group (nmol/mg protein·min or % of the control I_c_; means $\pm$ SE, n = 12).

^5^ ${IC}_{50}$ is the incubation time (h, parameter estimate $\pm$ SE, n = 72) required to reach the half maximal inhibition of the cellulase activity.

^6^Relative cellulase activity calculated as percentage (%, means $\pm$ SE, n = 12) of the control group (${I}_{c}$) measured at 0-min exposure to the combined gastric pH-3.5 and pepsin (274 U/mL) incubation.

Overall, these in vitro gastric acidic pH (pH = 3.5) with pepsin (274 U/mL) enzyme inhibition experiments shown that the GH5-p4818Cel5_2A activity was substantially reduced whereas the GH5-tCel5A1activity was marginally affected by the combined presence of the porcine physiological gastric acidic pH and pepsin. The GH5-tCel5A1 activity was sensitive to the gastric acidic pH and could lose more than 60% of its activity. The heat-treatment of GH5-tCel5a1 cellulase was shown detrimental to the GH5-tCel5A1 stability to the combined porcine physiological gastric acidic pH and pepsin.

### Experiment 3—These target endocellulases were not resistant to porcine pancreatic proteases in vitro

**Figure 12:**
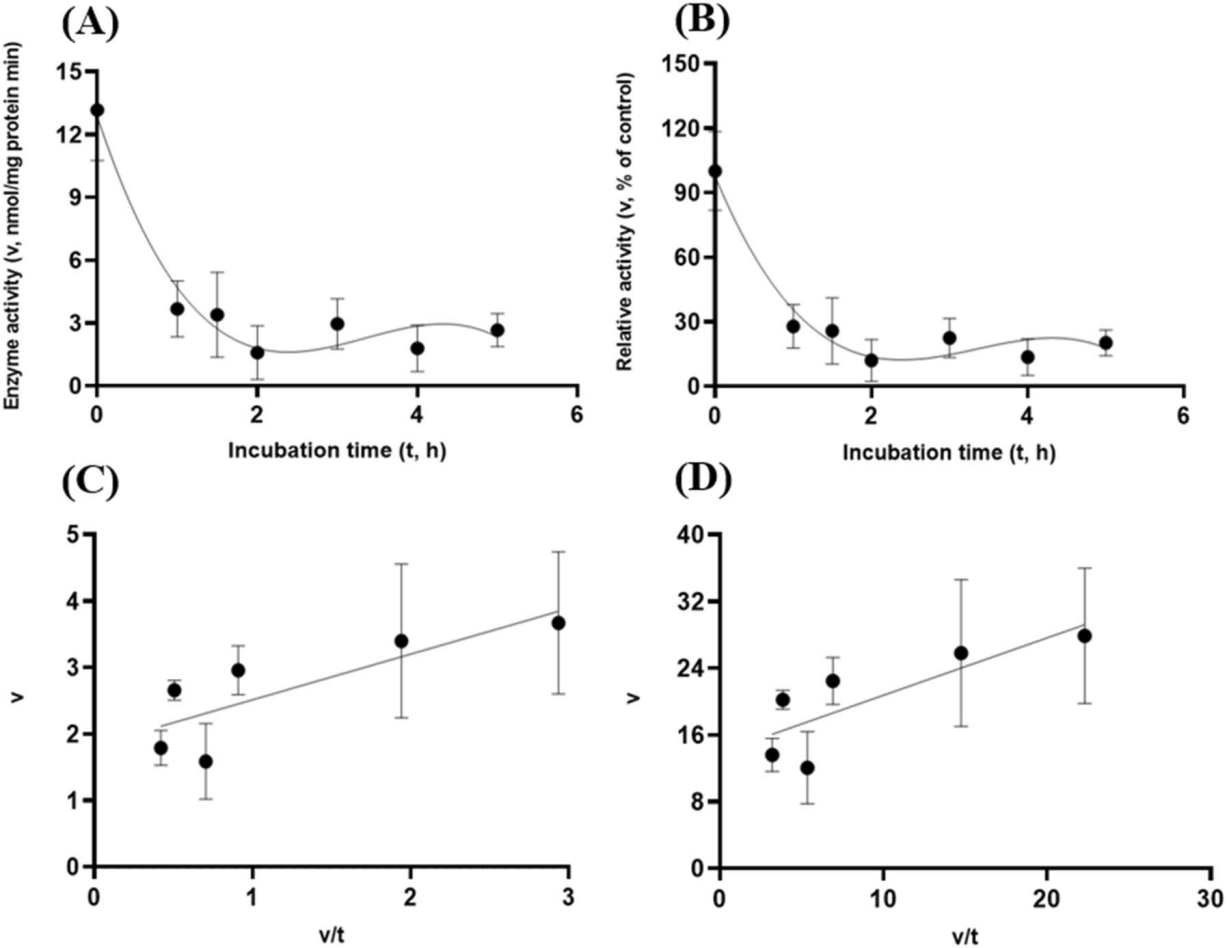
Experiment-3 of the intestinal pancreatic trypsin (78 U/mL) effects (with describing model parameter estimates $\pm \mathrm{SE})$ on GH5-tCel5A1 activity over a 5-h period after the intestinal pancreatic trypsin buffer being mixed with the GH5-tCel5A1enzyme preparation for incubation at the resulting mixture pH = 6.0–7.0 and trypsin (78 U/mL) prior to further incubation for measuring cellulase activity with the carboxymethyl cellulose (CMC) substrate buffer in presence of dithiothreitol (DTT) (5 mM) and N_2_ gas purging for 15 min at 37 °C. **(A)** Plot of the inhibition kinetic relationship between the GH5-tCel5A1 activity (v, mean $\pm \mathrm{SE},\mathrm{ n}=12\mathrm{ \,at\,each\,time\,point},$ nmol/mg protein•min) and incubation time (t, h) under the trypsin (78 U/mL) condition established according to the cubic response model as: $\mathrm{Y}=16.140\left(\pm 2.166\right)-13.659\left(\pm 3.342\right)\mathrm{x}+4.087\left(\pm 1.451\right){x}^{2}-0.378(\pm 0.176){x}^{3}$, *P* < 0.0001 for the intercept; *P* = 0.0001 for the linear parameter; *P* = 0.006 for the quadratic parameter estimate; and *P* = 0.035 for the cubic parameter, ${\mathrm{R}}^{2}=0.326$, n = 84; and alternatively, according to the linear regression model as: $\mathrm{y}=7.930\left(\pm 1.251\right)-1.459\left(\pm 0.405\right)\mathrm{x}$, *P* < 0.0001 for the intercept; and *P* = 0.001 for the slope, ${\mathrm{r}}^{2}=0.142$, n = 84; **(B)** Plot of the inhibition kinetic relationship between the relative GH5-tCel5A1 activity (v, mean $\pm \mathrm{SE},\mathrm{ n}=12\mathrm{ \,at\,each\,time\,point},$ % of the control group measured at I_c_ of 100.000 ± 18.294 at 0-min exposure to the trypsin-78-U/mL incubation) and incubation time (t, h) established according to the cubic response model: $\mathrm{Y}=122.6\left(\pm 16.458\right)-103.8\left(\pm 25.393\right)\mathrm{x}+31.050\left(\pm 11.026\right){x}^{2}-2.875(\pm 1.338){x}^{3}$, *P* < 0.0001 for the intercept*; P* = 0.0001 for the linear parameter estimate*; P* = 0.006 for the quadratic parameter estimate; and *P* = 0.035 for the cubic parameter estimate, ${\mathrm{R}}^{2}=0.326$, n = 84; and alternatively, according to the linear regression model established as: $\mathrm{y}=60.251\left(\pm 9.5\right)-11.091\left(\pm 3.077\right)\mathrm{x}$, *P* < 0.0001 for the intercept; and *P* = 0.001 for the slope; ${\mathrm{r}}^{2}=0.142$, n = 84; **(C**) the Eadie-Hofstee linear plot between the GH5-tCel5A1 enzyme activity (v, mean $\pm \mathrm{SE},\mathrm{ n}=12\mathrm{ \,at\,each\,data\,point},$ nmol/mg protein•min) and the v/t ratio established as: $\mathrm{y}=0.681\left(\pm 0.251\right)+1.639\left(\pm 0.090\right)\mathrm{x}$, *P* = 0.008 for the intercept; and *P* < 0.0001 for the slope, ${\mathrm{r}}^{2}=0.829$, n = 72; and **(D**) the Eadie-Hofstee linear plot between the relative GH5-tCel5A1 enzyme activity (v, mean $\pm \mathrm{SE},\mathrm{ n}=12\mathrm{ \,at\,each\,data\,point},$ % of the control group measured at I*c* of 100.000 ± 18.294) at 0-min exposure to the trypsin-78-U/mL incubation) incubation] and the v/t ratio established as $\mathrm{y}=5.173\left(\pm 1.904\right)+1.639\left(\pm 0.090\right)\mathrm{x}$, *P* = 0.008 for the intercept; and *P* < 0.0001 for the slope; ${\mathrm{r}}^{2}=0.829$, n = 72.

**Figure 13:**
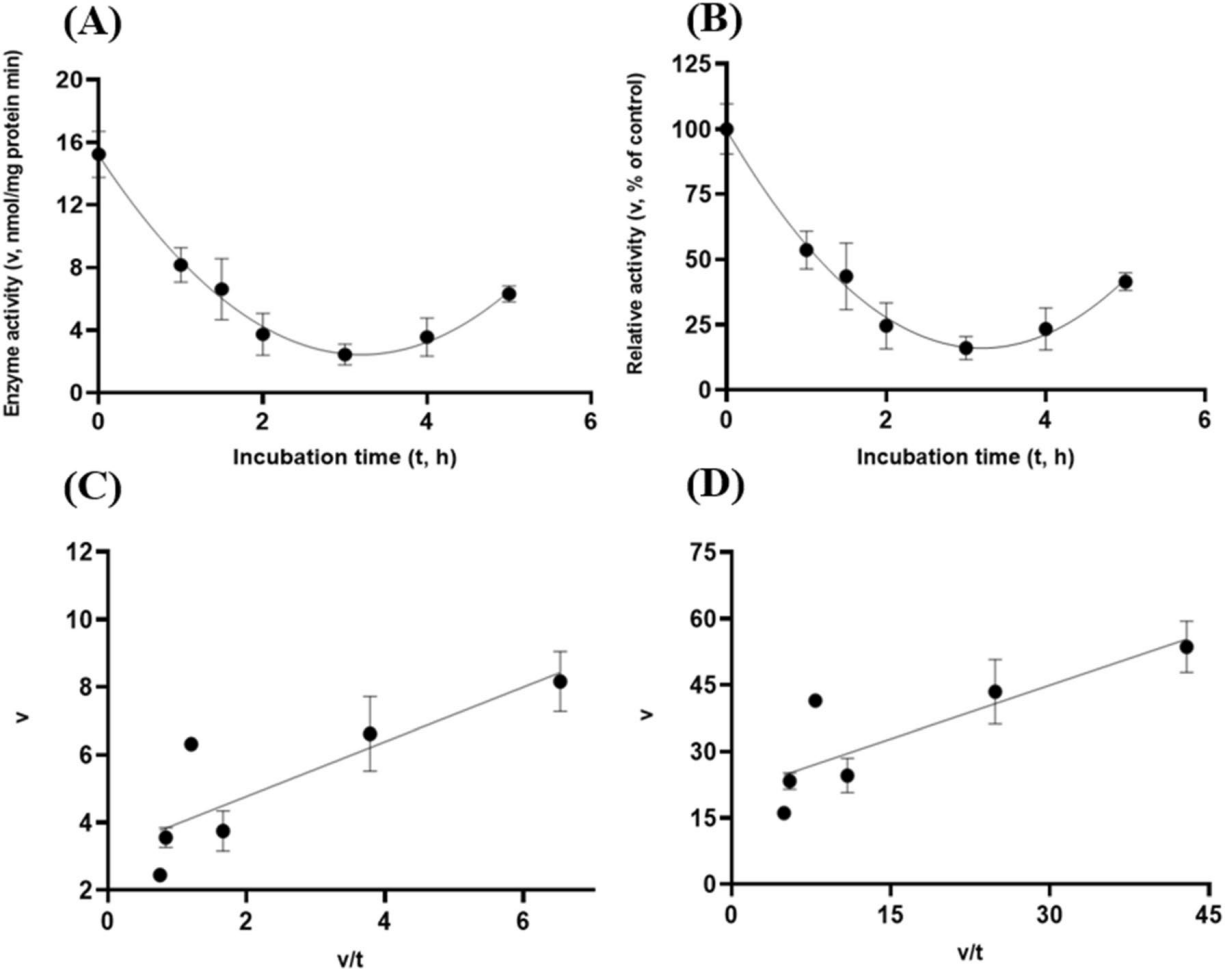
Experiment-3 of the intestinal pancreatic trypsin (78 U/mL) effects (with describing model parameter estimates $\pm \mathrm{SE})$ on heat-treated GH5-tCel5A1 activity over a 5-h period after the intestinal pancreatic trypsin buffer being mixed with the heat-treated GH5-tCel5A1 enzyme preparation for incubation at the resulting mixture pH = 6.0–7.0 and trypsin (78 U/mL) prior to further incubation for measuring cellulase activity with the carboxymethyl cellulose (CMC) substrate buffer in presence of dithiothreitol (DTT) (5 mM) and N_2_ gas purging for 15 min at 37 °C. **(A)** Plot of the inhibition kinetic relationship between the heat-treated GH5-tCel5A1 activity (v, mean $\pm \mathrm{SE},\mathrm{ n}=12\mathrm{ \,at\,each\,time\,point},$ nmol/mg protein•min) and incubation time (t, h) under the trypsin (78 U/mL) condition established according to the quadratic response model as: $\mathrm{Y}=17.408\left(\pm 1.388\right)-8.694\left(\pm 1.154\right)\mathrm{x}+1.259\left(\pm 0.198\right){x}^{2}$, *P* < 0.0001 for all parameter estimates; ${\mathrm{R}}^{2}=0.475$, n = 84; and alternatively according to the linear response model established as $\mathrm{y}=10.718\left(\pm 1.103\right)-1.599\left(\pm 0.357\right)\mathrm{x}$, *P* < 0.0001 for all parameter estimates, ${\mathrm{r}}^{2}=0.209$, n = 84; **(B)** Quadratic plot of the inhibition kinetic relationship between the relationship of relative heat-treated GH5-tCel5A1 activity (v, mean $\pm \mathrm{SE},\mathrm{ n}=12\mathrm{ \,at\,each\,time\,point},$ % of the control group measured at I_c_ of 100.000 ± 9.682) at 0-min exposure to the trypsin-78-U/mL incubation) and incubation time trypsin (78 U/mL) conditions established according to the quadratic response model as: $\mathrm{Y}=114.3\left(\pm 9.116\right)-57.093\left(\pm 7.581\right)\mathrm{x}+8.273\left(\pm 1.302\right){x}^{2}$, *P* < 0.0001 for all parameter estimates, ${\mathrm{R}}^{2}=0.475$, n = 84; and alternatively established according to the linear response model as $\mathrm{y}=70.388\left(\pm 7.242\right)-10.502\left(\pm 2.346\right)\mathrm{x}$, *P* < 0.0001 for all parameter estimates, ${\mathrm{r}}^{2}=0.210$, n = 84; **(C**) the Eadie-Hofstee linear plot between the heat-treated GH5-tCel5A1 enzyme activity (v, mean $\pm \mathrm{SE},\mathrm{ n}=12\mathrm{ \,at\,each\,data\,point},$ nmol/mg protein•min) and the v/t ratio established as $\mathrm{y}=1.918\left(\pm 0.353\right)+1.322\left(\pm 0.092\right)\mathrm{x}$, *P* < 0.0001 for all parameter estimates; ${\mathrm{r}}^{2}=0.749$, n = 72; and **(D**) the Eadie-Hofstee linear plot between the relative heat-treated GH5-tCel5A1 enzyme activity (v, mean $\pm \mathrm{SE},\mathrm{ n}=12\mathrm{ \,at\,each\,data\,point},$ % of the control group measured at *I*_*c*_ of 100.000 ± 9.682) at 0-min exposure to the trypsin-78-U/mL incubation) and the v/t ratio established as $\mathrm{y}=12.602\left(\pm 2.321\right)+1.322\left(\pm 0.092\right)\mathrm{x}$, *P* < 0.0001 for all parameter estimates; ${\mathrm{r}}^{2}=0.749$, n = 72.

**Figure 14:**
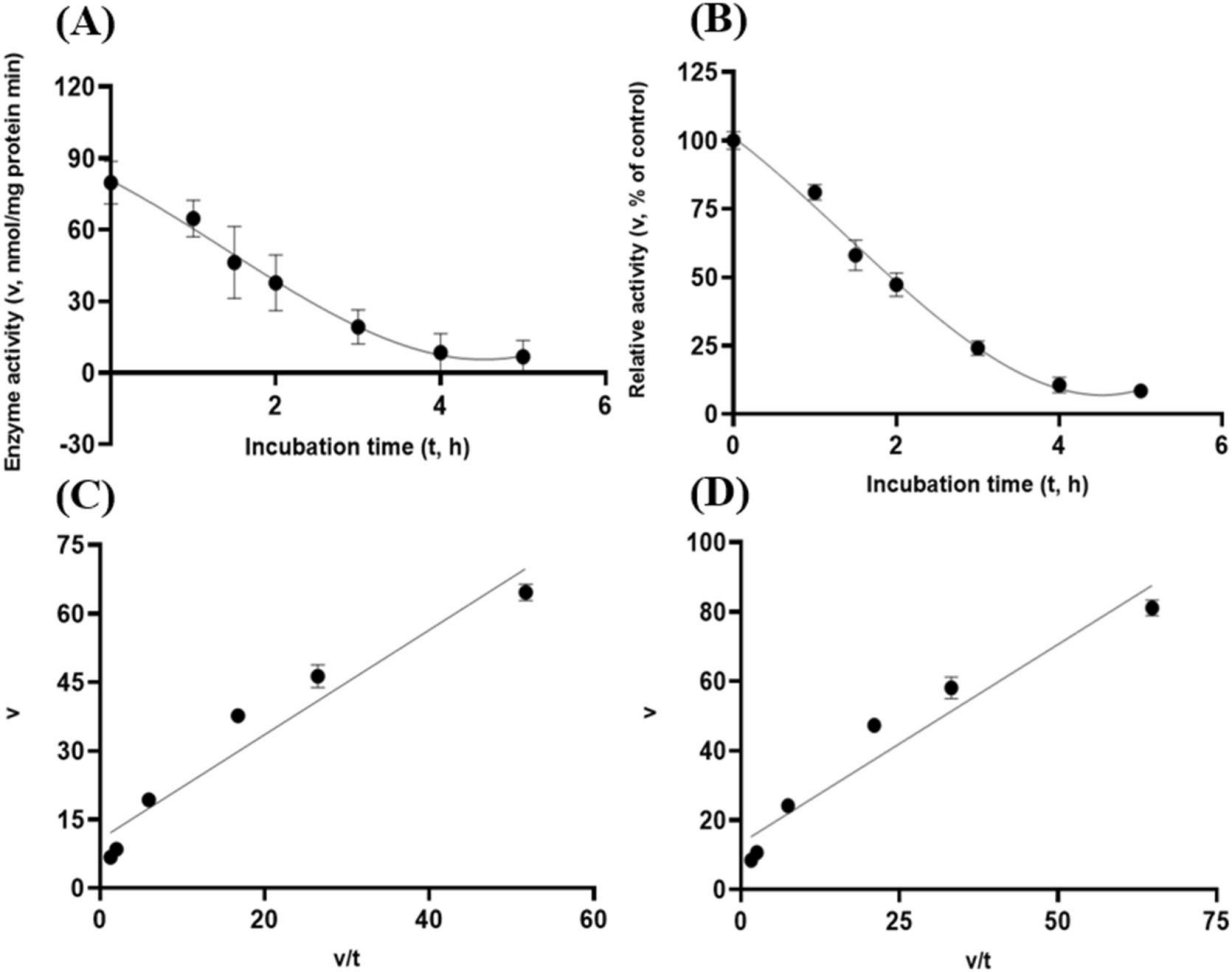
Experiment-3 of the intestinal pancreatic trypsin (78 U/mL) effects (with describing model parameter estimates $\pm \mathrm{SE})$ on GH5-p4818Cel5_2A activity over a 5-h period after the intestinal pancreatic trypsin buffer being mixed with the GH5-p4818Cel5_2A enzyme preparation for incubation at the resulting mixture pH = 6.0–7.0 and trypsin (78 U/mL) prior to further incubation for measuring cellulase activity with the carboxymethyl cellulose (CMC) substrate buffer in presence of dithiothreitol (DTT) (5 mM) and N_2_ gas purging for 15 min at 37 °C. **(A)** Plot of the inhibition kinetic relationship between the GH5-p4818Cel5_2A activity (v, mean $\pm \mathrm{SE},\mathrm{ n}=12\mathrm{ \,at\,each\,time\,point},$ nmol/mg protein•min) and incubation time (t, h) under the trypsin (78 U/mL) condition established according to the cubic response model as: $\mathrm{Y}=86.219\left(\pm 3.969\right)-17.601\left(\pm 6.123\right)\mathrm{x}+0.703(\pm 0.323){x}^{3}$, *P* < 0.0001 for the intercept; *P* = 0.005 for the linear parameter estimate*, P* = 0.232 for the quadratic parameter estimate; and *P* = 0.032 for the cubic parameter estimate; ${\mathrm{R}}^{2}=0.883$, n = 84; and alternatively according to the linear response model established as $\mathrm{y}=78.177\left(\pm 2.445\right)-15.559\left(\pm 0.792\right)\mathrm{x}$, *P* < 0.0001 for all parameter estimates, ${\mathrm{r}}^{2}=0.830$, n = 84; **(B)** Plot of the inhibition kinetic relationship between the relative GH5-p4818Cel5_2A activity (v, mean $\pm \mathrm{SE},\mathrm{ n}=12\mathrm{ \,at\,each\,time\,point},$ % of the control group measured at I_c_ of 100.000 ± 3.214) at 0-min exposure to the trypsin-78-U/mL incubation) and incubation time (t, h) under the trypsin (78 U/mL) condition established according to the cubic response model established as: $\mathrm{Y}=108.1\left(\pm 4.977\right)-22.075\left(\pm 7.679\right)\mathrm{x}+0.881(\pm 0.405){x}^{3}$, *P* < 0.0001 for the intercept*, P* = 0.005 for the linear parameter; *P* = 0.232 for the quadratic parameter and *P* = 0.032 for the cubic parameter; ${\mathrm{R}}^{2}=0.883$, n = 84; and according to the linear response model established as $\mathrm{y}=98.044\left(\pm 3.066\right)-19.513\left(\pm 0.993\right)\mathrm{x}$, *P* < 0.0001 for all parameter estimates, ${\mathrm{r}}^{2}=0.830$, n = 84; **(C**) the Eadie-Hofstee linear plot between the GH5-p4818Cel5_2A enzyme activity (v, mean $\pm \mathrm{SE},\mathrm{ n}=12\mathrm{ \,at\,each\,data\,point},$ µmol/mg protein•min) and the v/t ratio established as: $\mathrm{y}=9.762\left(\pm 1.158\right)+1.202\left(\pm 0.047\right)\mathrm{x}$, *P* < 0.0001 for all parameter estimates; ${\mathrm{r}}^{2}=0.906$, n = 72; and **(D**) the Eadie-Hofstee linear plot between the relative GH5-p4818Cel5_2A enzyme activity (v, mean $\pm \mathrm{SE},\mathrm{ n}=12\mathrm{ \,at\,each\,data\,point},$ % of the control group measured at *I*_*c*_ of 100.000 ± 3.214) at 0-min exposure to the trypsin-78-U/mL incubation) and the v/t ratio established as: $\mathrm{y}=12.244\left(\pm 1.453\right)+1.202\left(\pm 0.047\right)\mathrm{x}$, *P* < 0.0001 all parameter estimates, ${\mathrm{r}}^{2}=0.906$, n = 72.

These target enzymes’ stability within the small intestinal luminal conditions with resistance to the exocrine pancreatic trypsin (60 U/mL) was also investigated. In line with the preceding in vitro gastric stability experiments, the target enzyme samples were incubated with trypsin (60 U/mL) at pH 6.5 with the ${\mathrm{N}}_{2}$ gas purging treatment. Both GH5-tCel5A1 and GH5-p4818Cel5_2A exhibited cubic and linear patterns of responses (*P* < 0.05) between the enzyme activity and incubation time under the intestinal trypsin (60 U/mL) condition with a relatively lower coefficient of determination (r^2^) associated with the linear responses than with the cubic responses (R^2^) observed in Fig. 12A–B and Fig. 14A–B. However, the heat-treated tCel5A1 displayed quadratic and linear patterns of responses (*P* < 0.05) between the enzyme activity and incubation time under the small intestinal trypsin (60 U/mL at pH 6.5) condition with a relatively lower coefficient of determination (r^2^) associated with the linear responses than with the quadratic responses (R^2^) obtained (Fig. 13A–B).

These intestinal trypsin (60 U/mL at pH 6.5) inhibition kinetics over the 5-h period were obtained through conducting the Eadie-Hoftsee linear regression analyses as shown in Fig. 12C–D for GH5-tCel5A1; Fig. 13C–D for the heat-treated GH5-tCel5A1; and Fig. 14C–D for p4818Cel5_2A, respectively. These intestinal trypsin (60 U/mL at pH 6.5) inhibition kinetics are further summarized in Table 3 for all of the three target enzymes. With the *IC*_*50*_ = 1.64 and 1.32 h respectively observed for both GH5-tCel5A1 and the heat-treated tCel5A1 cellulases, their corresponding relative maximal enzyme activity inhibition (i.e., ${I}_{max}$) was estimated to be at 94.83 vs. 87.39%, showing subtle differences in these key inhibition kinetic endpoints between GH5-tCel5A1 and its heat-treated version of the GH5-tCel5A1 cellulase by the intestinal trypsin (60 U/mL) at pH 6.5. Furthermore, GH5-p4818Cel5_2A had an estimated relative maximal enzyme activity inhibition ${I}_{max}$ = 87.76% with the *IC*_*50*_ = 1.20 h by the intestinal trypsin (60 U/mL) at pH 6.5.

**Table 3:** Comparative summary of the Experiment-3 in vitro inhibition kinetics^1^ of GH5-tCel5A1, the heat-treated GH5-tCel5A1 and GH5-p4818Cel5_2A under the intestinal trypsin (74 U/mL) condition obtained through the Eadie-Hofstee linear regression analyses.

Item	Inhibition kinetic parameter estimates				
	${I}_{max}$ ^2^		${I}_{min}$ ^3^	${I}_{\mathrm{c}}$ ^4^	${IC}_{50}$ ^5^
**Cellulase activity (nmol/mg protein·min)**					
GH5-tCel5A1	12.48 $\pm 2.41$		0.68 $\pm 0.25$	13.16 $\pm 2.41$	1.64 $\pm 0.09$
Heated GH5-tCel5A1	3.26 $\pm 1.47$		1.92 $\pm 0.35$	5.17 $\pm 1.47$	1.32 $\pm 0.09$
GH5-p4818Cel5_2A	69.98 $\pm 2.56$		9.76 $\pm 1.16$	79.74 $\pm 2.56$	1.20 $\pm 0.05$
**Relative cellulase activity (% of the control)**^6^					
GH5-tCel5A1	$94.83\pm 18.29$		5.$17\pm 1.90$	100.00 $\pm 18.29$	1.64 $\pm 0.09$
Heated GH5-tCel5A1	87.39 $\pm 9.68$		12.60 $\pm 2.32$	100.00 $\pm 9.68$	1.32 $\pm 0.09$
GH5-p4818Cel5_2A	87.76 $\pm 3.21$		12.24 $\pm 1.45$	100.00 $\pm 3.21$	1.20 $\pm 0.05$

^1^Values are parameter estimates or means $\pm$ SE (n = 72) obtained through the Eadie-Hofstee regression linear analyses, representing the inhibition kinetic parameter estimates obtained from the three intestinal trypsin (74 U/mL) inhibition experiments with 4 replicates per time point in each experiment, as shown in Figs. 12, 13, 14.

^2^ ${I}_{max}$ is the maximal magnitude of inhibition in the cellulase activity (nmol/mg protein·min or % of the control, *I*_*c*_; means $\pm$ SE, n = 12) calculated according to Eq. (2).

^3^ ${I}_{min}$ is the minimal residual cellulase activity (nmol/mg protein·min or % of the control, parameter estimate $\pm$ SE, n = 72) under the intestinal trypsin (74 U/mL) inhibition condition obtained from Eq. (1).

^4^ ${I}_{c}$ is the mean cellulase activity of the control group (nmol/mg protein·min or % of the control *I*_*c*_; means $\pm$ SE, n = 12).

^5^ ${IC}_{50}$ is the incubation time (h, parameter estimate $\pm$ SE, n = 72) required to reach the half maximal inhibition of the cellulase activity.

^6^Relative cellulase activity calculated as percentage (%, means $\pm$ SE, n = 12) of the control group (${I}_{c}$) measured at 0-min exposure to the intestinal trypsin (74 U/mL) incubation.

**Figure 15:**
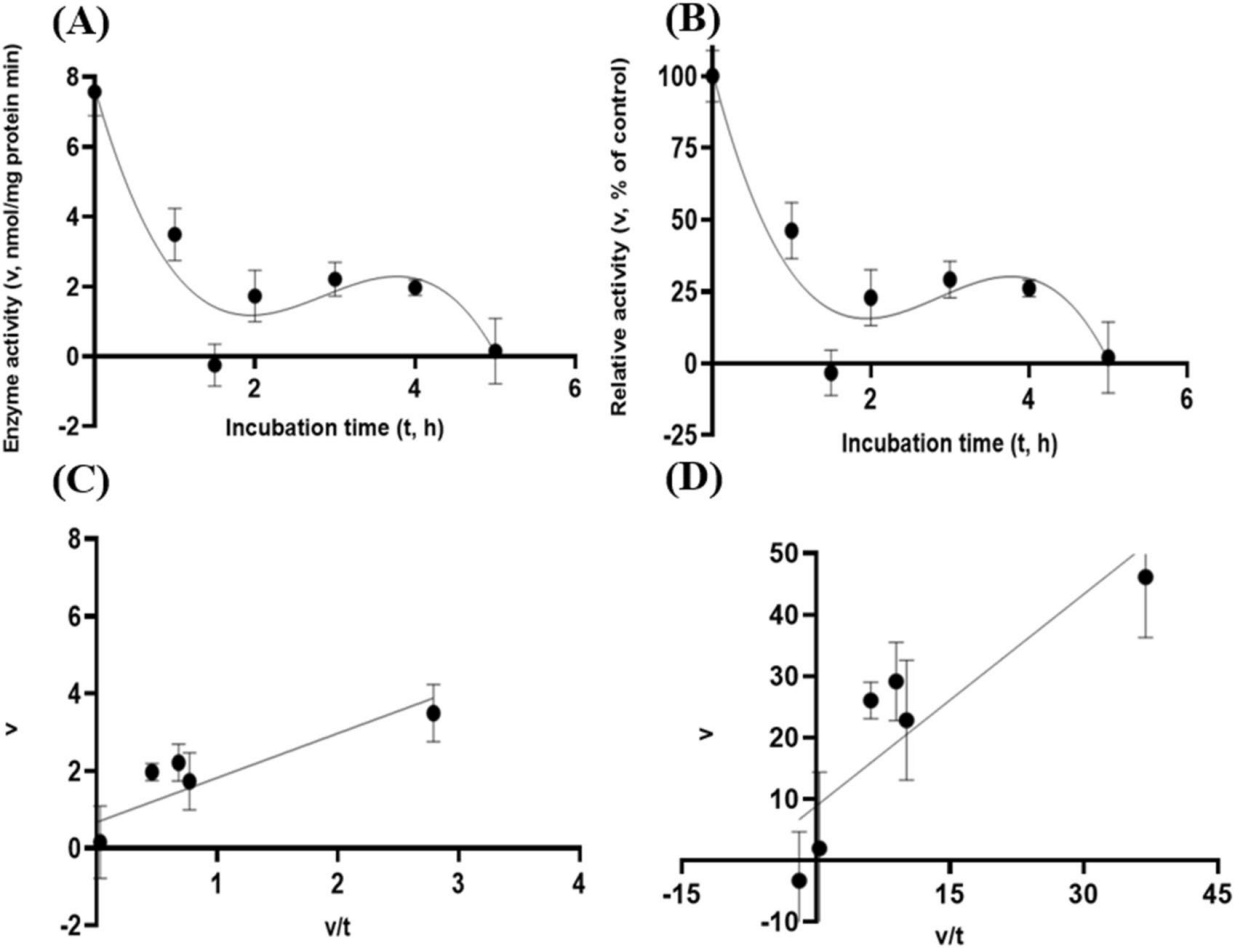
Experiment-3 of the intestinal pancreatic chymotrypsin (20 U/mL) effects (with describing model parameter estimates $\pm \mathrm{SE})$ on GH5-tCel5A1 activity over a 5-h period after the intestinal pancreatic chymotrypsin buffer being mixed with the GH5-tCel5A1 enzyme preparation for incubation at the resulting mixture pH = 6.0–7.0 and chymotrypsin (20 U/mL) prior to further incubation for measuring cellulase activity with the carboxymethyl cellulose (CMC) substrate buffer in presence of dithiothreitol (DTT) (5 mM) and N_2_ gas purging for 15 min at 37 °C. **(A)** Plot of the inhibition kinetic relationship between the GH5-tCel5A1 activity (v, mean $\pm \mathrm{SE},\mathrm{ n}=12\mathrm{ \,at\,each\,time\,point},$ nmol/mg protein•min) and incubation time (t, h) under the chymotrypsin (20 U/mL) condition established according to the cubic response model as: $\mathrm{Y}=9.908\left(\pm 0.983\right)-9.709\left(\pm 1.517\right)\mathrm{x}+3.416\left(\pm 0.659\right){x}^{2}-0.366(\pm 0.079){x}^{3}$, *P* < 0.0001 for all parameter estimates; ${\mathrm{R}}^{2}=0.469$, n = 84; and alternatively according to the linear response model as: $\mathrm{y}=4.828\left(\pm 0.609\right)-0.942\left(\pm 0.197\right)\mathrm{x}$, *P* < 0.0001 for all parameter estimates; ${\mathrm{r}}^{2}=0.224$, n = 84; **(B)** Plot of the inhibition kinetic relationship between the relative GH5-tCel5A1 activity (v, mean $\pm \mathrm{SE},\mathrm{ n}=12\mathrm{ \,at\,each\,time\,point},$ % of the control group measured at I_c_ of 100.000 ± 9.944) at 0-min exposure to the chymotrypsin-20-U/mL incubation) and incubation time (t, h) chymotrypsin according to the cubic response model established as: $\mathrm{Y}=130.8\left(\pm 12.982\right)-128.2\left(\pm 20.030\right)\mathrm{x}+45.105\left(\pm 8.698\right){x}^{2}-4.836(\pm 1.055){x}^{3}$, *P* < 0.0001 for all the parameter estimates; ${\mathrm{R}}^{2}=0.471$, n = 84; and alternatively according to the linear response model as: $\mathrm{y}=63.742\left(\pm 8.034\right)-12.439\left(\pm 2.602\right)\mathrm{x}$, *P* < 0.0001 for all parameter estimates; ${\mathrm{r}}^{2}=0.225$, n = 84; **(C**) the Eadie-Hofstee linear plot between the tCel5A1 enzyme activity (v, mean $\pm \mathrm{SE},\mathrm{ n}=12\mathrm{ \,at\,each\,data\,point},$ nmol/mg protein•min) and the v/t ratio established as: $\mathrm{y}=0.365\left(\pm 0.172\right)+1.592\left(\pm 0.115\right)\mathrm{x}$, *P* = 0.037 for the intercept; and *P* < 0.0001 for the slope; ${\mathrm{r}}^{2}=0.746$, n = 72; and **(D**) the Eadie-Hofstee linear plot between the relative GH5-tCel5A1 enzyme activity (v, mean $\pm \mathrm{SE},\mathrm{ n}=12\mathrm{ \,at\,each\,data\,point},$ % of the control group measured at *I*_*c*_ of 100.000 ± 9.944) at 0-min exposure to the chymotrypsin-20-U/mL incubation) and the v/t ratio established as: $\mathrm{y}=4.822\left(\pm 2.268\right)+1.592\left(\pm 0.115\right)\mathrm{x}$, *P* = 0.037 for the intercept; and *P* < 0.0001 for the slope, ${\mathrm{r}}^{2}=0.746$, n = 72.

**Figure 16:**
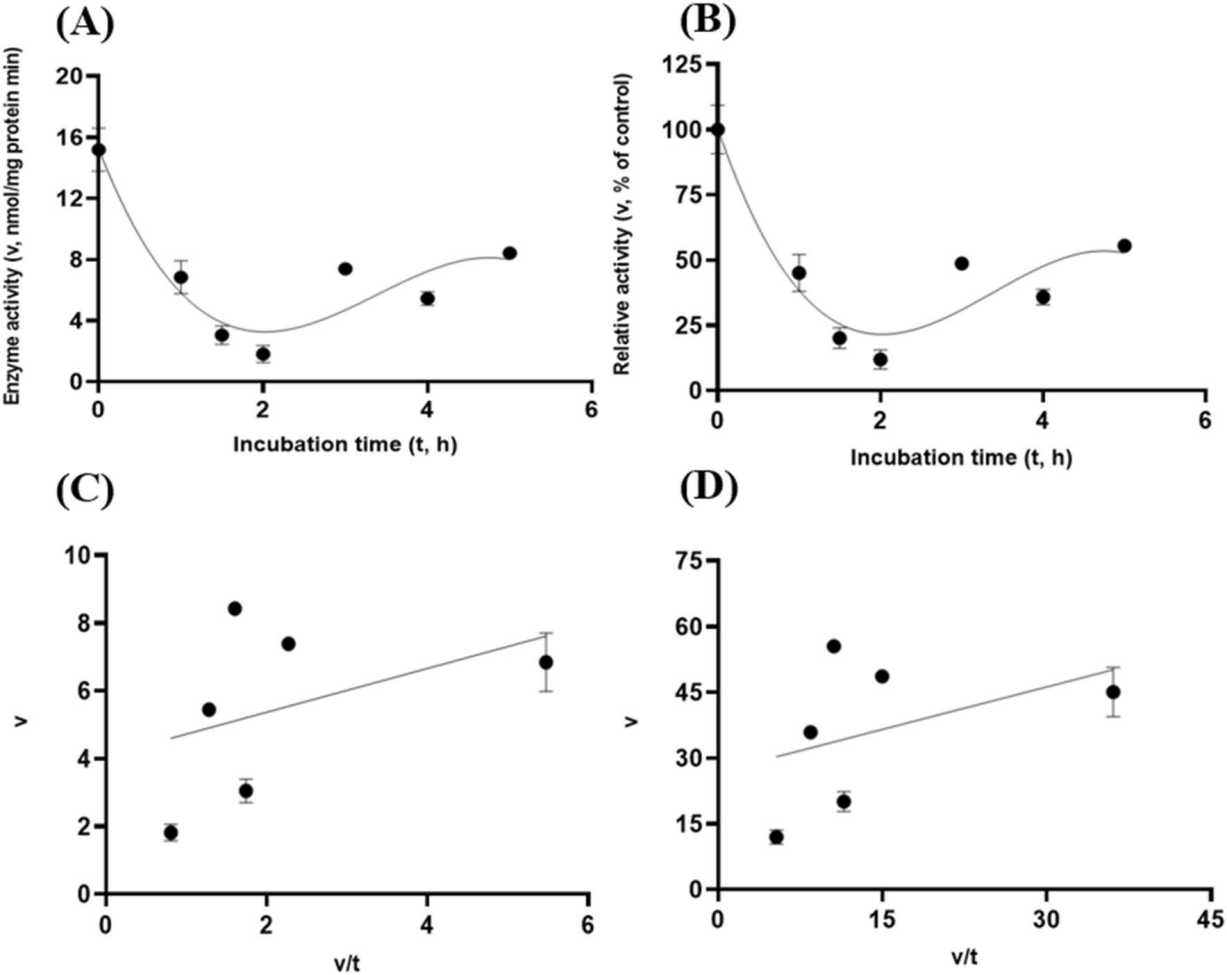
Experiment-3 of the intestinal pancreatic chymotrypsin (20 U/mL) effects (with describing model parameter estimates $\pm \mathrm{SE})$ on heat-treated GH5-tCel5A1 activity over a 5-h period after the intestinal pancreatic chymotrypsin buffer being mixed with the heat-treated enzyme preparation for incubation at the resulting mixture pH = 6.0–7.0 and chymotrypsin (20 U/mL) prior to further incubation for measuring cellulase activity with the carboxymethyl cellulose (CMC) substrate buffer in presence of dithiothreitol (DTT) (5 mM) and N_2_ gas purging for 15 min at 37 °C. **(A)** Plot of the inhibition kinetic relationship between the heat-treated GH5-tCel5A1 activity (v, mean $\pm \mathrm{SE},\mathrm{ n}=12\mathrm{ \,at\,each\,time\,point},$ nmol/mg protein•min) and incubation time (t, h) according to the cubic response model established as: $\mathrm{Y}=19.238\left(\pm 1.237\right)-16.580\left(\pm 1.908\right)\mathrm{x}+5.308\left(\pm 0.829\right){x}^{2}-0.487(\pm 0.101){x}^{3}$, *P* < 0.0001 for all parameter estimates; ${\mathrm{R}}^{2}=0.621$, n = 84; and alternatively according to the linear response model established as: $\mathrm{y}=8.428\left(\pm 0.994\right)-0.618\left(\pm 0.322\right)x$, *P* < 0.0001 for the intercept; and *P* = 0.058 for the slope; ${\mathrm{r}}^{2}=0.065$, n = 84; **(B)** Plot of the inhibition kinetic relationship between the relative heat-treated GH5-tCel5A1 activity (v, mean $\pm \mathrm{SE},\mathrm{ n}=12\mathrm{ \,at\,each\,time\,point},$ % of the control group measured at I_c_ of 100.000 ± 9.259) at 0-min exposure to the chymotrypsin-20-U/mL incubation) and incubation time (t, h) established according to the cubic response model as: $\mathrm{Y}=126.7\left(\pm 8.146\right)-109.2\left(\pm 12.569\right)\mathrm{x}+34.965\left(\pm 5.458\right){x}^{2}-3.210(\pm 0.662){x}^{3}$, *P* < 0.0001 for all the parameter estimates; ${\mathrm{R}}^{2}=0.622$, n = 84; and alternatively according to the linear response model as: $\mathrm{y}=55.523\left(\pm 6.547\right)-4.072\left(\pm 2.121\right)\mathrm{x}$, *P* < 0.0001 for the intercept; and *P* = 0.058 for the slope; ${\mathrm{r}}^{2}=0.065$, n = 84; **(C**) the Eadie-Hofstee linear plot between the heat-treated GH5-tCel5A1 enzyme activity (v, mean $\pm \mathrm{SE},\mathrm{ n}=12\mathrm{ \,at\,each\,data\,point},$ nmol/mg protein•min) and the v/t ratio established as: $\mathrm{y}=3.337\left(\pm 0.424\right)+0.998\left(\pm 0.142\right)\mathrm{x}$, *P* < 00001 for all parameter estimates, ${\mathrm{r}}^{2}=0.421$, n = 72; and **(D**) the Eadie-Hofstee linear plot between the relative heat-treated GH5-tCel5A1 enzyme activity (v, mean $\pm \mathrm{SE},\mathrm{ n}=12\mathrm{ \,at\,each\,data\,point},$ % of the control group measured at *I*_*c*_ of 100.000 ± 9.259) at 0-min exposure to the chymotrypsin-20-U/mL incubation) and the v/t ratio established as: $\mathrm{y}=21.988\left(\pm 2.793\right)+0.998\left(\pm 0.142\right)\mathrm{x}$, *P* < 0.0001 for all parameter estimates,${\mathrm{r}}^{2}=0.421,$ n = 72.

**Figure 17:**
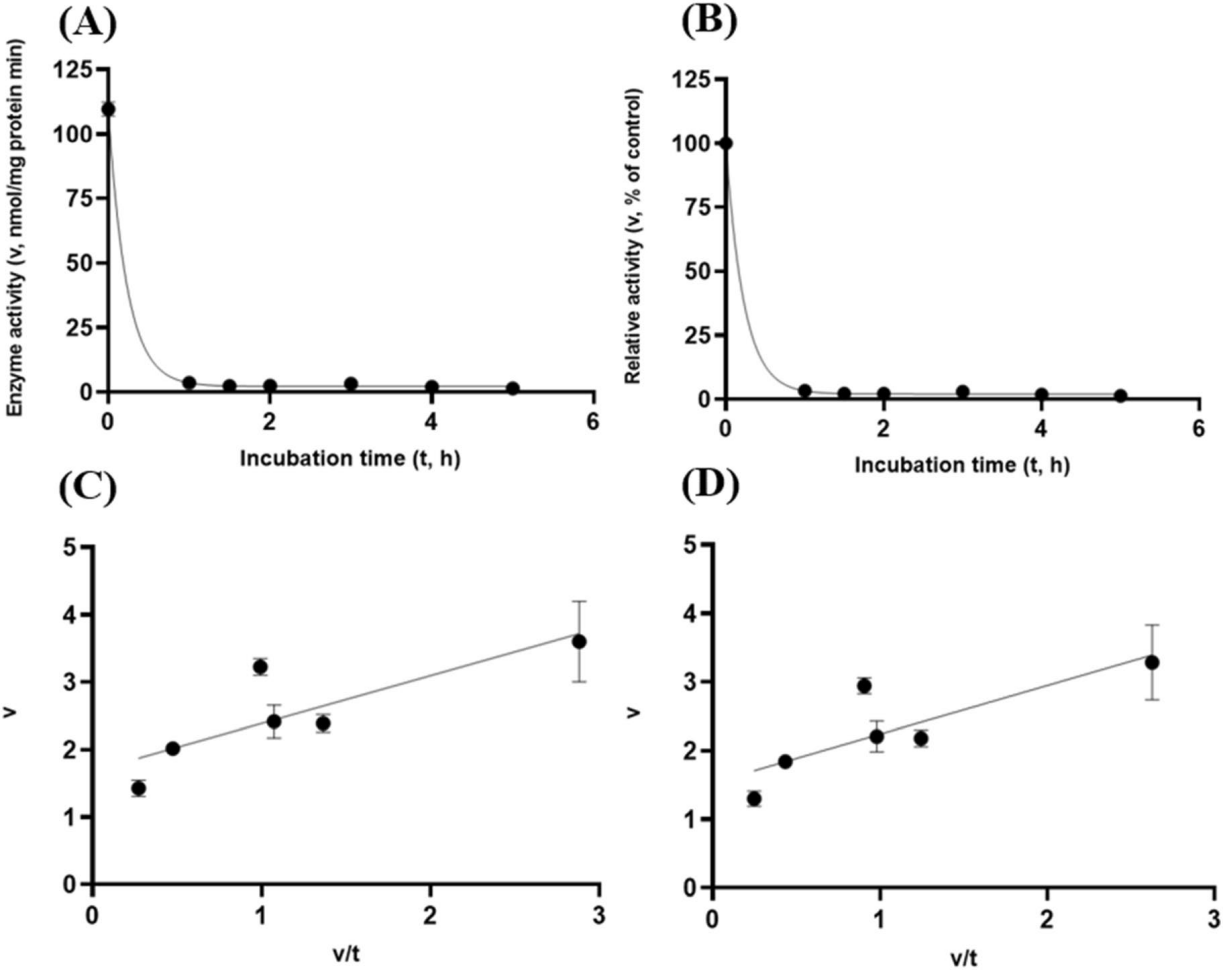
Experiment-3 of the intestinal pancreatic chymotrypsin (20 U/mL) effects (with describing model parameter estimates $\pm \mathrm{SE})$ on GH5-p4818Cel5_2A activity over a 5-h period after the intestinal pancreatic chymotrypsin buffer being mixed with the heat-treated enzyme preparation for incubation at the resulting mixture pH = 6.0–7.0 and chymotrypsin (20 U/mL) prior to further incubation for measuring cellulase activity with the carboxymethyl cellulose (CMC) substrate buffer in presence of dithiothreitol (DTT) (5 mM) and N_2_ gas purging for 15 min at 37 °C. **(A)** Plot of the inhibition kinetic relationship between the GH5-p4818Cel5_2A activity (v, mean $\pm \mathrm{SE},\mathrm{ n}=12\mathrm{ \,at\,each\,time\,point},$ nmol/mg protein•min) and incubation time (t, h) established according to the exponential response model as: $Y=250.7\left(\pm 16.314\right){\mathrm{e}}^{\left[-3.263\left(\pm 0.255\right)\mathrm{x}\right)]}$, *P* < 0.0001 for all parameter estimates; ${\mathrm{R}}^{2}=0.989$, n = 84; and alternatively according to the linear response model established as: $\mathrm{y}=52.465\left(\pm 6.366\right)-13.503\left(\pm 2.062\right)\mathrm{x}$, *P* < 0.0001 for all the parameter estimates; ${\mathrm{r}}^{2}=0.348$, n = 84; **(B)** Plot of the inhibition kinetic relationship between the relative GH5-p4818Cel5_2A activity (v, mean $\pm \mathrm{SE},\mathrm{ n}=12\mathrm{ \,at\,each\,time\,point},$ % of the control group measured at I_c_ of 100.000 ± 2.455) at 0-min exposure to the chymotrypsin-20-U/mL incubation) and incubation time (t, h) established according to the exponential response model as: $Y=228.7\left(\pm 14.878\right){\mathrm{e}}^{\left[-3.263\left(\pm 0.255\right)\mathrm{x}\right)]}$, *P* < 0.0001 for all the parameter estimates; ${\mathrm{R}}^{2}=0.989$, n = 84; and alternatively according to the linear response model established as: $\mathrm{y}=47.856\left(\pm 5.807\right)-12.317\left(\pm 1.881\right)\mathrm{x}$, *P* < 0.0001 for all parameter estimates; ${\mathrm{r}}^{2}=0.348$, n = 84; **(C**) the Eadie-Hofstee linear plot between the GH5-p4818Cel5_2A enzyme activity (v, mean $\pm \mathrm{SE},\mathrm{ n}=12\mathrm{ \,at\,each\,time\,point},$ nmol/mg protein•min) and the v/t ratio established as: $\mathrm{y}=1.121\left(\pm 0.171\right)+1.196\left(\pm 0.099\right)\mathrm{x}$, *P* < 00001 for all parameter estimates, ${\mathrm{r}}^{2}=0.682$, n = 72; and **(D**) the Eadie-Hofstee linear plot between the relative GH5-p4818Cel5_2A enzyme activity (v, mean $\pm \mathrm{SE},\mathrm{ n}=12\mathrm{ \,at\,each\,data\,point},$ % of the control group measured *I*_*c*_ of 100.000 ± 2.455) at 0-min exposure to the chymotrypsin-20-U/mL incubation) and the v/t ratio established as: $\mathrm{y}=1.023\left(\pm 0.156\right)+1.196\left(\pm 0.099\right)\mathrm{x}$, *P* < 0.0001 for all parameter estimates;${\mathrm{r}}^{2}=0.682,$ n = 72.

Lastly, the target enzymes’ stability within the small intestinal luminal conditions with resistance to the exocrine pancreatic chymotrypsin (20 U/mL) was also investigated. Similar to the preceding in vitro trypsin stability experiments, these target enzyme samples were incubated with chymotrypsin (20 U/mL at pH 6.5) with the ${\mathrm{N}}_{2}$ gas purging treatment. GH5-tCel5A1 demonstrated cubic and linear patterns of responses (*P* < 0.05) (Fig. 15A–B); while the heat-treated GH5-tCel5A1 cellulase only shown the cubic pattern of response (*P* < 0.05) (Fig. 16A–B) under the small intestinal chymotrypsin (20 U/mL at pH 6.5) condition. Furthermore, GH5-p4818Cel5_2A yielded exponential decay curve and linear patterns of responses (*P* < 0.05) with a much lower coefficient of determination (r^2^) associated with the linear response than with the strong exponential decay response (R^2^) analyzed (Fig. 17A–B).

These small intestinal chymotrypsin (20 U/mL at pH 6.5) inhibition kinetics over the 5-h period were further carried out via the Eadie-Hofstee linear regression analysis as shown in Fig. 15C–D for tCel5A1; Fig. 16C–D for the heat-treated tCel5A1; and Fig. 17C–D for GH5-p4818Cel5_2A, respectively. These small intestinal chymotrypsin (20 U/mL at pH 6.5) inhibition kinetics are further summarized in Table 4, for all of these three target enzymes. With the *IC*_*50*_ = 1.59 and 1.00 h respectively observed for both GH5-tCel5A1 and the heat-treated GH5-tCel5A1 cellulases, their corresponding relative maximal enzyme activity inhibition (i.e., ${I}_{max}$) was estimated to be at 95.18 vs. 78.01%, clearly demonstrating differences in these key inhibition kinetic endpoints between GH5-tCel5A1 and its heat-treated version of the GH5-tCel5A1 cellulase by the small intestinal chymotrypsin (20 U/mL at pH 6.5). Furthermore, GH5-p4818Cel5_2A had an estimated relative maximal enzyme activity inhibition ${I}_{max}$ = 98.98% with the *IC*_*50*_ = 1.20 h by the small intestinal chymotrypsin (20 U/mL at pH 6.5).

**Table 4:** Comparative summary of the Experiment-3 in vitro inhibition kinetics^1^ of GH5-tCel5A1, the heat-treated GH5-tCel5A1 and GH5-p4818Cel5_2A under the intestinal Chymotrypsin (20 U/mL) condition obtained through the Eadie-Hofstee linear regression analyses.

	Inhibition kinetic parameter estimates				
Item	${I}_{max}$ ^2^		${I}_{min}$ ^3^	${I}_{c}$ ^4^	${IC}_{50}$ ^5^
Cellulase activity (nmol/mg protein·min)					
GH5-tCel5A1	7.21 $\pm 0.68$		0.37 $\pm 0.17$	7.57 $\pm 0.68$	1.59 $\pm 0.12$
Heated GH5-tCel5A1	11.84 $\pm 1.41$		3.34 $\pm 0.42$	15.18 $\pm 1.41$	0.99 $\pm 0.14$
GH5-p4818Cel5_2A	108.51 $\pm 2.69$		1.12 $\pm 0.17$	109.63 $\pm 2.69$	1.19 $\pm 0.09$
Relative cellulase activity (% of the control)^6^					
GH5-tCel5A1	95.18 $\pm 8.94$		4.82 $\pm 2.27$	100.00 $\pm 8.94$	1.59 $\pm 0.12$
Heated GH5-tCel5A1	78.01 $\pm 9.26$		21.98 $\pm 2.79$	100.00 $\pm 9.26$	1.00 $\pm 0.14$
GH5-p4818Cel5_2A	98.98 $\pm 2.46$		1.02 $\pm 0.16$	100.00 $\pm 2.46$	1.20 $\pm 0.09$

^1^Values are parameter estimates or means $\pm$ SE (n = 72) obtained through the Eadie-Hofstee regression linear analyses, representing the inhibition kinetic parameter estimates obtained from the three chymotrypsin (20 U/mL) inhibition experiments with 4 replicates per time point in each experiment, as shown in Figs. 15, 16, 17.

^2^ ${I}_{max}$ is the maximal magnitude of inhibition in the cellulase activity (nmol/mg protein·min or % of the control, means $\pm$ SE, n = 12) calculated according to Eq. (2).

^3^ ${I}_{min}$ is the minimal residual cellulase activity (nmol/mg protein·min or % of the control *I*_*c*_; parameter estimate $\pm$ SE, n = 72) under the intestinal chymotrypsin (20 U/mL) inhibition condition obtained from Eq. (1).

^4^ ${I}_{c}$ is the mean cellulase activity of the control group (nmol/mg protein·min or % of the control, means $\pm$ SE, n = 12).

^5^ ${IC}_{50}$ is the incubation time (h, parameter estimate $\pm$ SE, n = 72) required to reach the half maximal inhibition of the cellulase activity.

^6^Relative cellulase activity calculated as percentage (%, means $\pm$ SE, n = 12) of the control group (*I*_*c*_) measured at 0-min exposure to the intestinal chymotrypsin (20 U/mL) incubation.

In summary, these in vitro inhibition experiments with the small intestinal trypsin (60 U/mL) and chymotrypsin (20 U/mL) at pH 6.5 shown that all of the three tested target cellulases, i.e., GH5-p4818Cel5_2A; tCel5A1 and the heat-treated tCel5A1 would substantially lose their activities when exposed to these two exocrine pancreatic proteases. Whereas heat-treatment of GH5-tCel5a1 cellulase was shown to partially enhance the GH5-tCel5A1 resistance to the exocrine pancreatic proteases at pH 6.5 in vitro.

## Discussion

Our primary objectives of this study were to investigate enzyme stability for the two recently discovered mono-modular processive endoglucanases of GH5-tCel5A1 and GH5-p4818Cel5_2A under the mimicked in vitro porcine gut physiological conditions. More specifically, we overexpressed both GH5-tCel5A1 and GH5-p4818Cel5_2A cellulases in the endotoxin-free *E. coli* strain of CLEARCOLI BL21 (DE3)^40^. The over-expression of tCel5A1 in the CLEARCOLI BL21 (DE3) cell was further confirmed in the cell homogenate, the Ni–NTA gel purified GH5-Cel5A1 cellulase and the heat-processed version of this GH5-Cel5A1 cellulase (Fig. 2). As revealed in Fig. 2, the purified GH5-Cel5A1 gel bands on lanes #4 and 8 would represent the target cellulase GH5-tCel5A1 at about 37 kDa, which is consistent with the previously reported GH5-tCel5A1 cellulase at about 37 kDa^25^. The over-expression of the GH5-p4818Cel5_2A in the CLEARCOLI BL21 (DE3) cell was confirmed in the cell homogenate and the Ni–NTA gel purified GH5-p4818Cel5_2A cellulase via the SDS-PAGE analyses as reported in our previous studies^11^. Furthermore, after initial enzyme activity assays (data not shown here), we observed a significant reduction in enzyme activity in the purified GH5-Cel5A1 cellulase sample in contrast to the anticipation that the Ni–NTA purification would dramatically enhance this target cellulase activity^41^. Thus, rather than using the Ni–NTA purified target GH5-tCel5A1 and GH5-p4818Cel5_2A cellulases, we chosen to conduct this enzyme stability study with the freshly homogenized and flash-frozen CLEARCOLI BL21 (DE3) cell homogenates with respectively over-expressed target GH5-tCel5A1 and GH5-p4818Cel5_2A processive endoglucanases by using the CMC as a substrate.

We used the 3-D models for prediction of Cys residues in both GH5-tCel5A1 and GH5-p4818Cel5_2A (Fig. 1A and B). Our results showed the presence of four Cys residues in GH5-p4818Cel5_2A and one Cys residue in GH5-tCel5A1. In addition, all of the predicted Cys residues with a free thiol group were located near their catalytic sites, respectively (Fig. 1A and B). For designing the depletion of auto-oxidation treatment group, all of the three target crude enzyme samples were prepared by using the cell lysis buffer containing the artificial anti-oxidant DTT at 5 mM. The cell lysis buffer, the enzyme incubation buffers and the enzyme incubation mixtures were then thoroughly purged with pressured pure ${\mathrm{N}}_{2}$ gas immediately prior to the cell lysis and the enzyme incubation procedures for depletion of the airborne O_2_ as an inexpensive test system as previously reported^42^. Clearly, our time course experimental results by using CMC and Avicel collectively shown that both GH5-tCel5A1 and GH5-p4818Cel5_2A were associated with predicted Cys residues and likely contained one or more free -HS group located near their catalytic sites, thus both enzymes were susceptible to auto-oxidation by the airborne O_2_ and displayed very poor enzyme stability (Figs. 3 and 5). These results are consistent with the fact that the GH5-tCel5A1 gene was originated from the extremely thermophilic *Thermotoga maritima*^25,43^, belonging to the *Thermotoga* genus of strictly anaerobic bacteria^44^ while GH5-p4818Cel5_2A was screened out of the porcine distal ileal-cecal microbiome of a highly anaerobic gut environment^11^. Furthermore, these results are in line with the study by Takata et al.^37^ in showing reduced ${\mathrm{Ca}}^{2+}$-calmodulin dependent protein kinase IV activity due to the oxidation of the thiol groups located on Cys residues. It should also be pointed out that in the study by Wang et al.^11^, the anti-oxidant DTT at 5 mM was used in all of their buffers for reducing auto-oxidation and maintaining the GH5-p4818Cel5_2A stability. While the concept that Cys residues with free -SH group(s) on active sites of the enzyme proteins are susceptible to auto-oxidation thus leading to protein structural and functional property changes has been well reviewed^38^, to the best of our knowledge, this study would be among the first group of studies in demonstrating that the presence of HS-containing Cys residues in catalytic sites negatively affected exogenous fibre degradation enzyme stability due to auto-oxidation. Considering that there is significant and varying degree of oxygenation within the upper gastrointestinal tract^45,46^, auto-oxidation by airborne O_2_ should be recognized as one of the major intrinsic factors limiting exogenous fibre enzyme stability, shelf-life and in vivo efficacy in food animal and potentially human nutrition applications.

We next examined GH5-tCel5A1, the heat-treated Gh5-tCel5A1 and GH5-p4818Cel5_2A for their stability under the mimicked typical in vitro porcine gastric pH (3.5) and pepsin (274 U/mL) conditions (Figs. 6, 7, 8, 9, 10, 11). The above designed gastric physiological conditions have reflected our understanding and efforts made for mimicking the three key aspects of (i) the variable porcine gastric acidic pH, typically at pH 2–4.5 during the feeding^26^; (ii) an upper porcine gastric pepsin activity at about 266 U/mL digesta fluid^1^; and (iii) the porcine gastric retention time of feed digesta within 2–5 h^26^. We carried out the in vitro gastric enzyme stability experiments within the literature reported porcine gastric retention time frame (0–5 h) (Fig. 6, 7, 8, 9, 10, 11). The Eadie-Hofstee inhibition kinetic analyses further enabled us to estimate the two essential inhibition kinetic parameter estimates, including the maximal magnitude of inhibition in these target cellulases’ activity (*I*_*max*_, nmol/mg protein·min or % of the control groups) and the incubation time (${IC}_{50}$, h) required to reach the half maximal inhibition of these cellulases’ activity (Tables 1 and 2). The results of our gastric stability Experiment-1 suggest that GH5-tCel5A1 activity and stability were only marginally affected by about 13%, whereas the thermal-processed GH5-tCel5A1 and GH5-p4818Cel5_2A respectively lost up to 49 and 68% of their activities under the gastric acidic pH and pepsin conditions within the conceived gastric retention time frame (i.e., 2 x ${IC}_{50}$ = 1.4, 1.7 and 2.1 h). In comparison, various in vitro gastric conditions have been reported in the literature. Qin et al.^47^ observed that Cel5A endoglucanase of *Trichoderma reesei* lost 10% of its activity under a pH range of 4.6–5.0. Zhao et al.^33^ characterized a β-1,4-glucanase for its stability under their gastric acidic pH conditions and resistance to proteolysis by pepsin. Ohno et al.^35^ examined the stability of an avian chitinase in the presence of pepsin-C at pH 2.0 and found the enzyme resistant to pepsin at 1 h of incubation. Thus, gastric physiological conditions would differentially affect exogenous and endogenous fibre enzyme stability. The incubation medium acidic pH, pepsin activity and incubation time should be designed to closely simulate in vivo gastric physiological conditions of the concerned animal species to be relevant when characterizing enzyme stability in vitro.

In order to continue the characterization of these target cellulases under physiologically relevant small intestinal luminal conditions, the small intestinal enzyme stability Experiments-2 and 3 in this study were designed for simulating again three key aspects of (i) the variable porcine small intestinal luminal pH, typically at pH 6–8 during the feeding^26^; (ii) the upper porcine small intestinal luminal trypsin (at 60 U/mL) and chymotrypsin (at 20 U/mL) activities in digesta fluid, respectively^21^; and (iii) the porcine small intestinal retention time of feed digesta to be within 2–9 h^26^. We also carried out the in vitro small intestinal enzyme stability Experiments-2 and 3 within the literature reported porcine intestinal retention time frame (0 – 5 h) (Figs. 12, 13, 14, 15, 16, 17) in combination with the Eadie-Hofstee inhibition kinetic analyses in obtaining the key inhibition kinetic endpoints of *I*_*max*_ and ${IC}_{50}$ (Tables 3 and 4). The results of our intestinal trypsin stability Experiment-2 indicate that GH5-tCel5A1, the thermal-processed GH5-tCel5A1 and GH5-p4818Cel5_2A would lose 85 to 88% of their activity under the intestinal pH and the trypsin conditions within the conceived small intestinal retention time frame (i.e., 2 x ${IC}_{50}$ = 3.4, 2.6 and 2.2 h). The intestinal chymotrypsin stability Experiment-3 data suggest that GH5-tCel5A1 and GH5-p4818Cel5_2A lost the majority (95 to 99%) of their original activities whereas the thermal-processed GH5-tCel5A1 lost up to 78% of its activity under the intestinal pH and the chymotrypsin conditions within the conceived small intestinal retention time frame (i.e., 2 x ${IC}_{50}$ = 2.4, 3.2 and 2.0 h). Exogenous fibre enzyme resistance to pancreatic proteases has been reported previously. Zhao et al.^33^ further investigated the stability of β-1,4-glucanase to proteolysis by trypsin and observed that 94% of its original cellulase activity was retained after 1 h of incubations with trypsin; however, no ${IC}_{50}$ value was estimated in the study. In our previous studies, Wang et al.^11^ reported that GH5-p4818Cel5_2A would lose almost all of its activity at the end of the 6-h incubation period; however, trypsin (5000 U/mL) and chymotrypsin (at 200 U/mL) activities used in that study’s incubations were much higher than these respective pancreatic proteases’ activity levels (at 60 and 20 U/mL) in the previously reported digesta fluid of the porcine gut lumen^21^. Hence, intestinal physiological conditions could differentially affect exogenous fibre enzyme stability. The incubation medium pH, the exocrine pancreatic proteases’ activity levels and incubation time should be designed to closely mimic in vivo small intestinal physiological conditions of the concerned animal species to be relevant when characterizing fiber enzyme stability in vitro.

Overall, both GH5-tCel5A1 and GH5-p4818Cel5_2A are mono-modular, processive and multi-functional^7,11,25^. As further demonstrated in this study, GH5-tCel5A1, the thermal-processed version of the GH5-tCel5A1 and GH5-p4818Cel5_2A all displayed significant activities in hydrolyzing the soluble and crystalline cellulosic substrates (Figs. 3, 4, 5). The thermophilic property of GH5-tCel5A1 is an important feature for feed industrial applications as many commercial compound feed manufacturers require higher pelleting & conditioning temperatures that can potentially denature mesophilic exogenous fibre enzymes^7,48,49^. Whereas the mesophilic GH5-p4818Cel5_2A can be potentially supplemented in commercial diets via exogenous enzyme coating and post-pelleting liquid exogenous enzyme product spray^50^. Auto-oxidation of both GH5-tCel5A1 and GH5-p4818Cel5_2A by airborne O_2_, as shown in this study (Figs. 3 and 5), is linked to the presence of Cys residue(s) in their catalytic sites, which further reflects the strictly anaerobic bacteria origins of these two enzyme genes and potentially limits their enzyme stability, shelf-life and efficacy. Although site-specific mutagenesis may be feasible to replace Cys residues in the catalytic sites to potentially modulate the auto-oxidation associated enzyme stability concern, this genetic engineering strategy may be limiting since studies have shown that these catalytic site Cys residues can play essential functional roles such as thermostability^51^. Apparently, thermal-treatment of the hyper-thermophilic GH5-tCel5A1 was shown effective to eliminate the auto-oxidation associated stability concern for this enzyme (Figs. 3 and 4). However, the thermal-treated GH5-tCel5A1 became less resistant to the combined gastric acidic pH and pepsin (Table 2) and was still susceptible to intestinal trypsin and chymotrypsin (Tables 3 and 4). Although site-specific mutagenesis-based enzyme engineering may be possible to mitigate these gastric-small intestinal conditions (i.e., acidic pH and proteases)-associated enzyme stability concerns, mutagenesis of multiple AA residues on the catalytic sites of these cellulases may prove to be really challenging. Alternatively, organic polymer coating for encapsulation of target exogenous enzymes has been recognized to be effective for by-passing the stomach and the small intestinal conditions that may be detrimental to target enzyme stability^46,52^. And this strategy may also be effective to minimize these enzymes’ exposure to airborne O_2_. Therefore, organic polymer coating-based encapsulation may serve as a practical post-fermentation strategy to address the auto-oxidation and the gastrointestinal stability concerns and help deliver these target exogenous fibre enzymes to the porcine distal small intestine and the large intestine as the suitable sites.

In summary, we have demonstrated, with both GH5-tCel5A1 and GH5-p4818Cel5_2A, that recombinant exogenous cellulases with the predicted presence of catalytic site Cys residues were susceptible to auto-oxidation by airborne O_2_ and were less stable, thus potentially limiting the enzyme shelf-life and in vivo efficacy. We have further shown that GH5-tCel5A1, the thermal-processed GH5-tCel5A1 and GH5-p4818Cel5_2A were not resistant to acidic pH and proteases, thus these exogenous cellulases were not stable under the mimicked in vitro porcine gastric-small intestinal environmental conditions. Our results also suggest that thermal-treatment combined with organic polymer coating for the thermophilic GH5-tCel5A1 and organic polymer coating for GH5-p4818Cel5_2A for these cellulases’ enzyme encapsulation will need to be further developed to enable both cellulases as potentially efficacious exogenous fibre enzymes for the pig nutrition and global pork production commercial applications.

## Materials and methods

### Three-dimensional modelling

Functionality of endocellulases is largely due to their unique 3-D structures^53^. In addition, substrate specificity and mode of action of various glycosyl hydrolases such as GH5-tCel5A1 and GH5-p4818Cel5_2A are due to their 3-D structures^7,52^. The structure and functionality of GH5-tCel5A1 had been predicted and resolved in details in previous studies with the PDB# 3MMW^25,54^. In addition, GH5-tCel5A1 is a c-terminal truncated GH5-Cel5A through deletion of the 10 amino acids from its C-terminus^25^. Thus, the structure model of GH5-tCel5A1 was further predicted using 3MMW as a template. Previous 3-D modelling of GH5-p4818Cel5_2A by Wang et al.^11^. had demonstrated the presence a long tunnel-like active site topology for p4818Cel5_2A^11^. The presence of Cys residues in the enzyme catalytic sites would lead to potential susceptibility to auto-oxidation^55^. Thus, both GH5-tCel5A1 and GH5-p4818Cel5_2A cellulases were further modelled with a focus on predicted Cys residues on their respective catalytic sites using the SWISS-MODEL online server (https://swissmodel.expasy.org/) using the crystal structure of a homologous cellulase as a template (PDB#3MMW) for tCel5A1 and (PDB#1E5J) for GH5-p4818Cel5_2A based on the work by Wang et al.^11^ Afterwards, these predicted 3-D cellulases were analyzed and visualized by using PYMOL2.4.1.

### Protein expression

The AA sequence of tCel5A1 (GenBank #:AAD36816.1) was obtained from the original research work by Basit and Akhtar^25^. The nucleotide coding sequence of GH5-tCel5A1 was codon-optimized for over-expression in *E. Coli* and was commercially synthesized by Integrated DNA technologies as a gene block (Coralville, Iowa, USA). The GH5-tCel5A1 gene block was then ligated into the vector p15TV-L (accession number EF456736) and fused in frame with an N-terminal His-tag for generating p15TV-L-tCel5A1 as described by Wang et al.^11^. The construct was then verified by DNA sequencing at the University of Guelph Laboratory Service Molecular Super Center. This plasmid construct was then transformed into the CLEARCOLI BL21 (DE3) electrocompetent cells using the standard protocols by following our previously reported work by Wang et al.^11^.

CLEARCOLI BL21 (DE3) cells harboring the over-expression construct of p15TV-L/tCel5A1 were inoculated into 100 mL of Luria–Bertani (LB) stock solution (Thermo Scientific, Rockford, IL) along with 100 μL of ampicillin (100 μg /mL) on d 1 of growth^25^. The culture was then grown over night at 37 °C with shaking at 200 rpm in the New Brunswick™ Innova® 44 incubator (Eppendorf Canada Ltd, Mississauga, ON, Canada). On d 2, four flasks of 1-L of the LB stock solution were made with 1 mL of ampicillin (100 μg/mL) and 10 mL of culture grown previously overnight. Cultures were then grown overnight at 37 °C with shaking at 200 rpm on the same incubator previously mentioned. Enzyme expression induction was done on d 3 by lowering the culture temperature to 18 °C and adding 1 mL of 0.5-M isopropyl-β-D-thiogalactopyranoside (IPTG). Cultures were then left to grow overnight at 18 °C with shaking at 200 rpm. The cultured cells were harvested on d 4 via centrifugation at 3000 rpm for 15 min in the swinging bucket style rotor (29 cm radius, Beckman Coulter J6-MI centrifuge (Beckman Coulter Inc., Brea, CA). Cell pellets were then frozen at − 80 °C overnight. Lastly, sonication was done on the cell suspension on d 5. Cell pellets were resuspended in about 20 mL of a lysis buffer containing 0.3 M of NaCl, 5% glycerol, 5 mM DTT, with or without this depending upon the experimental design) and 0.05 M sodium HEPES at pH 7.0. Cells in resuspension were further disrupted by sonication and the lysed cell homogenates or crude extracts were frozen at − 20 °C for further use.

The construction of a metagenomic expression plasmid library via metagenomic screening, sequence and phylogenetic analyses for GH5-p4818Cel5_2A was performed as described by Wang et al.^11^. The gene encoding the mature form of the GH5-p4818Cel5_2A cellulase was amplified using PCR and these fragments were fused in frame with N-terminal His tag to create pET28a-GH5-p4818Cel5_2A using standard protocols^11^. This construct was then verified by DNA sequencing at the University of Guelph Laboratory Service Molecular Super Center. This plasmid construct was then transformed into CLEARCOLI BL21 (DE3) using our previously reported standard protocols^11^.

CLEARCOLI BL21 (DE3) cells harboring the over-expression construct of pET28a/p4818Cel5_2A were inoculated into 100 mL of the LB stock solution (Thermo Scientific, Rockford, IL) along with 100 μL of kanamycin (50 μg/mL) on d 1 of growth^11^. The culture was then grown over night at 37 °C with shaking at 200 rpm in the New Brunswick™ Innova® 44 incubator (Eppendorf Canada Ltd, Mississauga, ON, Canada). On d 2, four flasks of 1 L of the LB stock solution were made with 1 mL of kanamycin (50 μg/mL) and 10 mL of culture grown previously overnight. Cultures were then grown overnight at 37 °C with shaking at 200 rpm on the same incubator previously mentioned. Enzyme expression induction was done on d 3 by lowering temperatures to 15 °C and adding 1 mL of 0.5 M of IPTG. Cultures were then left to grow overnight at 15 °C with shaking at 200 rpm. The culture cells were harvested on d 4 via centrifugation at 4,200 rpm for 15 min with the same Beckman centrifuge described above. Cell pellets were then frozen at − 80 °C overnight. Lastly, sonication was done on d 5. Cell pellets were resuspended in about 20 mL of a lysis buffer containing 0.3 M NaCl, 5% glycerol, 5 mM DTT and 0.05 M sodium HEPES at pH 7.0. Cells in resuspension were further disrupted by sonication and the lysed cell homogenates or crude extracts were frozen at − 20 °C till further use. For comparing the airborne auto-oxidation impact, an alternative above lysis buffer was made without including 5 mM DTT.

### Protein assay and target endocellulases’ protein purification and SDS-PAGE analysis

Cell lysates and purified enzyme preparations were analysed for protein content by the BIO-RAD assay. To establish a calibration curve for protein concentration, a bovine serum albumin (BSA)-based protein gradient was established by using the BIO-RAD dye. All samples from both the BSA-based gradient standards and enzyme samples were corrected with a set of blank sample reading containing distilled and de-ionized H_2_0. The three cell lysate samples of tCel5A1, the heat-treated GH5-tCel5A1 and GH5-p4818Cel5_2A were diluted at 100 times of dilution and then used for the protein assay with optical density (OD) reading at 595 nm using the BioTek Synergy H1 microplate reader (BioTek Winooski, VT, USA).

In order to create a heat-treated version of the GH5-tCel5A1, the GH5-tCel5A1 cell lysate crude extract samples were heated at 65 °C for 20 min in a shaking-water bath. Upon completion, the heated samples were centrifuged at 3000 rpm for 10 min. In order to obtain purified GH5-tCel5A1 and GH5-p4818Cel5_2A in following our previously established protocols from another similar E. COLI BL21 (λDE3) cell system^11^, GH5-tCel5A1, the heat-treated GH5-tCel5A1 and GH5-p4818Cel5_2A cell lysates were first filtered through a 0.45-µm syringe filter and mixed with Ni–NTA resin for 40 min at 4 °C with gentle stirring. Afterwards, the mixture was then poured into a column for separation with affinity chromatography by washing with 300 mL of a buffer containing 300 mM NaCl, 50 mM HEPES and 20 mM midazole at pH 7.0. The bound enzyme proteins of GH5-tCel5A1, the heat-treated GH5-tCel5A1 and GH5-p4818Cel5_2A were eluted with the same buffer but with a higher concentration of imidazole at 250 mM.

Crude cell lysate samples of GH5-tCel5A1, the heat-treated GH5-tCel5A1 and GH5-p4818Cel5_2A along with the purified tCel5A1 and the heat-treated tCel5A1 enzyme preparations, were all denatured by heating at 100 °C for 5 min. Enzyme samples (3 µL) were loaded into Mini-PROTEAN® TGX Stain-Free™ Precast Gels (Bio-Rad Laboratories, Hercules, CA, USA). Heat treatment for the GH5-p4818Cel5_2A cellulases was not tested and analyzed due to the known lower thermostability of the GH5-p4818Cel5_2A reported from our previous study^11^. Protein bands in respective samples were separated using the SDS-PAGE electrophoresis and photographed with a stain-free imaging system (Bio-Rad Laboratories, Hercules, CA, USA)^11^.

### Experiment 1—Target Cellulases’ susceptibility to auto-oxidation by airborne O_2_ in vitro

In order to investigate the susceptibility of these endocellulases’ GH5-tCel5A1, the heat-treated GH5-tCel5A1 and GH5-p4818Cel5_2A to auto-oxidation by airborne O_2_, all three crude enzyme preparations were incubated with the CMC and Avicel substrate solutions to monitor endocellulases’ activity for up to 90 and 180 min, respectively at 37 °C. Only crude cell lysate enzyme preparation samples of GH5-tCel5A1, heat-treated GH5-tCel5A1 and GH5-p4818Cel5_2A were used for these assays in experiment 1. For the auto-oxidation control group, all three crude enzyme samples were prepared by using the cell lysis buffer not containing any artificial anti-oxidant DTT; and the cell lysis buffer and enzyme incubation buffers and enzyme incubation mixture were not purged with ${\mathrm{N}}_{2}$ gas immediately prior to cell lysis and enzyme incubation procedures for depletion of airborne O_2_ as previously reported^42^. On the contrary for the auto-oxidation depletion treatment group, all three crude enzyme samples were prepared by using the cell lysis buffer containing the artificial anti-oxidant DTT at 5 mM; and the cell lysis buffer and enzyme incubation buffers, enzyme incubation mixture and its headspace (i.e., the empty space above incubation mixture) were well purged with pressured pure ${\mathrm{N}}_{2}$ gas immediately prior to cell lysis and enzyme incubation procedures for removal of the airborne O_2_.

Substrate solutions of 1.25% CMC or 1.25% Avicel were made by adding 1.25 g of either CMC or Avicel with or without 5 mM DTT (dependent upon the treatment groups) into 100 mL of the 315-mOsm Krebs–Henseleit buffer solution containing 120 mM NaCl, 8.8 mM KCl, 1.2 mM MgS ${\mathrm{O}}_{4}$, 1.2 mM K ${\mathrm{H}}_{2}{\mathrm{PO}}_{4}$, 25 mM of NaHC ${\mathrm{O}}_{3}$ and 1.5 mM of ${\mathrm{CaCl}}_{2}$–2 ${\mathrm{H}}_{2}$ 0. The pH of the substrate solution was then brought down to 6.0 by addition of a 2-M HCl solution for meeting the reported optimal pH for both enzymes^11,25^. To be consistent with the substrate solutions, multiple aliquots of the 1.25% CMC and 1.25% Avicel substrate buffers were frozen for storage at − 20 °C till use.

All of three cell lysate-based crude enzyme preparations for the CMC substrate-based time course experiments were also further diluted by fourfold in containing protein contents at 8.06, 4.23 and 12.28 mg/mL for GH5-tCel5A1, heat-treated GH5-tCel5A1 and GH5-p4818Cel5_2A, respectively, with the 315-mOsm Krebs–Henseleit buffer, whereas the cell lysate-based crude enzyme preparations were directly used in the Avicel substrate-based susceptibility to auto-oxidation time course experiments without further dilution. Prior to the time course experiments, all enzyme preparation samples and the substrate solutions were pre-warmed in a shaking-water bath at 37 °C for 30 min. Afterwards, 0.050 mL of diluted enzyme preparation samples was incubated with 0.450 mL of the 1.25%-CMC or 1.25%-Avicel substrate solution.

In order to establish linearity of the planned time course experiments, GH5-tCel5A1, the heat-treated GH5-tCel5A1 and GH5-p4818Cel5_2A cellulase preparations, each at 0.050 mL, were incubated with 0.450 mL of either the 1.25%-CMC or the 1.25%-Avicel substrate solution containing or without 5 mM DTT for the period of 0 to 30 min at 37 °C with or without ${\mathrm{N}}_{2}$ purging treatment. For the CMC substrate-based time course experiments, enzyme incubations were carried out for a total of 90 min with samples being taken out at 0, 7.5, 15, 30, 45, 60, 75 and 90 min, respectively. For the Avicel substrate-based time course experiments, enzyme incubations were conducted for a total of 180 min with samples being taken out at 0, 30, 60, 120 and 180 min, respectively.

For the auto-oxidation depletion treatment group, immediately after initiation of the enzyme incubations, the sample incubation Eppendorf tube headspace was quickly purged with ${\mathrm{N}}_{2}$ gas in a fume hood then each tube was placed in the shaking-water bath at 100 rpm/h and at 37 °C. When respectively designated incubation time was reached for each sample tube, 1.500 mL of the 3,5-dinitrosalicylic acid (DNS) solution was added into each incubation tube for terminating the enzyme incubations and for estimating reducing sugar content by our previously reported procedures^11^. Samples were subsequently heated at 100 °C on a heating-block for 8 min and were cooled down to room temperature afterwards. Finally, 0.250 mL of the cooled and well mixed sample was then pipetted into a 96-well microplate for absorbance readings at 540 nm using a BioTek Synergy H1 microplate reader (BioTek Winooski, VT, USA).

### Experiment 2—Target Cellulases’ susceptibility to the porcine gastric pH and pepsin in vitro

To examine the stability of our target endocellulases of GH5-tCel5A1, the heat-treated GH5-tCel5A1 and GH5-p4818Cel5_2A under a typical porcine gastric pH alone condition and the combined gastric pH with pepsin (e.g., 274 U/mL) conditions, the diluted enzyme preparations (by fourfold), each at 0.050 mL, containing protein contents at 8.06, 4.23 and 12.28 mg/mL for GH5-tCel5A1, the heat-treated GH5-tCel5A1 and GH5-p4818Cel5_2A, respectively, were incubated at 37 °C after thoroughly mixing 0.450 mL of the 1.25%-CMC substrate solution with a blank control buffer (0.170 mL) as the control group and either a gastric-bicarbonate buffer at pH of 3.5 (0.170 mL) or a gastric-bicarbonate buffer with pepsin (355 U/mL buffer) (0.0284 g pepsin powder/20 mL buffer; 250,000 U/g P700-25G porcine pepsin powder, Sigma/Aldrich) and pH adjusted at 3.5 (0.170 mL). Porcine gastric pH during feeding was reviewed to be at about 2 to 4.5 in pigs^26^. This experiment adopted the upper porcine gastric pepsin activity at 274 U/mL based on the report by Woyengo et al.^56^. In addition, retention time of gastric digesta was reviewed to be within 2–5 h in pigs^26^. Thus, we designed this in vitro porcine gastric stability experiment to be at the gastric pH of 3.5 and a test duration for up to 5 h at the pepsin activity of 274 U/mL incubation mixture.

All target cellulases’ samples were diluted by fourfold using the the 315-mOsm Krebs–Henseleit buffer. The diluted enzyme preparation samples, the 1.25%-CMC substrate solution and the gastric-bicarbonate buffers (with and without pepsin at 274 U/mL) were all pre-warmed in a 37 °C shaking-water bath for 30 min prior to being used for the designed experiments. All afore-mentioned buffers and solutions were thoroughly purged with ${\mathrm{N}}_{2}$ gas to deplete dissolved airborne O^2^ prior to being used for the designed experiments^42^.

The gastric-bicarbonate buffer stock was made with 50 mM NaHC ${\mathrm{O}}_{3}$, 75 mM N ${\mathrm{a}}_{2}$ C ${\mathrm{O}}_{3}$, 5 mM MgC $\mathrm{l}$.6 ${\mathrm{H}}_{2}$ O, 10 mM Trizma-HCl, 10 mM HEPES and 5 mM DTT and the final mixture pH at 3.5 adjusted by using the 2-M HCl solution. The gastric-bicarbonate buffer with pepsin at 355 U/mL was made by using the above gastric-bicarbonate buffer with the addition of the porcine pepsin powder (0.0284 g of the porcine pepsin powder per 20 mL of the buffer; 250,000.0 U/g of the P700-25G porcine pepsin solid powder, Sigma/Aldrich with the enzyme activity U defined by the vendor) to anticipate the pepsin activity at 274 U/mL after mixing with the diluted enzyme preparations. The 1.25%-CMC substrate solution was made to contain 5 mM DTT with the 315-mOsm Krebs–Henseleit buffer and the final mixture pH at 6.15 adjusted by using the 2-M HCl solution. All the gastric stability experimental enzyme incubation mixtures (each at 0.500 mL) had pH within 6.0–7.0 when the 1.25%-CMC substrate solution (each at 0.280 mL) was mixed with the enzyme preparations (each at 0.050 mL) and the gastric-bicarbonate buffer (each at 0.170 mL), which was within the reported optimal activity pH ranges for these target cellulases^11,25^.

For each batch of the gastric stability experiments, 0.050 mL of diluted enzyme preparations was mixed with 0.170 mL of the gastric-bicarbonate buffer (with or without pepsin at 274 U/mL) to initiate the incubation experiment. Immediately, the sample tube headspace was purged with ${\mathrm{N}}_{2}$ gas and the enzyme tubes were incubated in a shaking water-bath at 37 °C for their respective time periods of 0 (the positive control group), 60, 90, 120, 180, 240 and 300 min, respectively. After the 5-h in vitro gastric stability incubation phase, each sample was then further mixed with 0.280 mL of the 1.25%-CMC substrate solution and the pH values of each incubation sample were recorded and were ensured to be within the optimum pH range (6.0–7.0) of the enzymes^11,25^. The headspace of each sample tube was quickly purged again with ${\mathrm{N}}_{2}$ gas and samples were incubated at 37 °C in a shaking water-bath for 15 min based on the previous linearity time course data measured with the 1.25%-CMC substrate solution. To terminate the reducing sugar reaction and begin the colour reaction, 1.5 mL of the DNS solution was added and heated at 100 °C for 8 min on a heating block. Upon completion, samples were well mixed, cooled and absorbances were read on the microplate reader at 540 nm.

A blank control group was also designed by addition of each of the diluted enzyme preparations (each at 0.050 mL) being incubated with the gastric-bicarbonate buffer (with or without pepsin at 274 U/mL) (each at 0.170 mL) for 300 min. At the completion of the 5-h in vitro gastric stability incubation phase, the blank control group was mixed with 0.280 mL of the 1.25%-CMC substrate solution and the mixture pH was quickly recorded. Immediately after, 1.500 mL of the DNS solution was added to inhibit the target enzyme activity and the samples were heated at 100 °C for 8 min on a heating block and the blank absorbances were read at 540 nm using the microplate reader for the in vitro gastric stability Experiment-2 sample blank corrections.

### Experiment 3—Cellulases’ susceptibility to the porcine intestinal trypsin and chymotrypsin in vitro

To further examine the stability of GH5-tCel5A1, the heat-treated GH5-tCel5A1 and GH5-p4818Cel5_2A in terms of resistance to the exocrine pancreatic proteases under intestinal conditions, we designed two additional experiments looking at in vitro resistance to trypsin and chymotrypsin present in incubation mixtures. In order to investigate intestinal stability characteristics, the target enzymes’ samples were incubated with an intestinal-bicarbonate buffer with either trypsin or chymotrypsin and then incubated with the 1.25%-CMC substrate at 37 °C for measuring endocellulase activity afterwards.

The small intestinal pH is typically at pH 6.0 to 8.0 with retention time of feed digesta in the small intestine to be within 2–9 h in pigs as compiled by Kidder and Manners^26^. In addition, these experiments adopted a previously reported upper porcine small intestinal trypsin activity at about 60 U/mL and chymotrypsin activity at about 20 U/mL, as reported by Fang et al.^21^. Thus, we designed these experiments to last for a total of 5 h with the intestinal-bicarbonate buffers at pH 6.5. In addition, the intestinal-bicarbonate buffers were made to anticipate trypsin activity at 60 U/mL incubation mixture and chymotrypsin activity at 20 U/mL incubation mixture, respectively.

The intestinal-bicarbonate buffer stocks were made with 50 mM NaHC ${\mathrm{O}}_{3}$, 75 mM N ${\mathrm{a}}_{2}$ C ${\mathrm{O}}_{3}$, 5 mM MgC $\mathrm{l}$ 6 ${\mathrm{H}}_{2}$ 0, 10 mM Trizma-HCl, 10 mM HEPES and 5 mM DTT and pH at 6.5 adjusted with the 2-M HCl solution. The intestinal-bicarbonate buffer with trypsin at 60 U/mL was further made by using the same intestinal-bicarbonate buffer above with the addition of 78 U/mL porcine trypsin powder (0.0039 g of the porcine trypsin powder per 50 mL of the buffer; 1000,000.0 U/g of the T4799-10G porcine trypsin powder, Sigma/Aldrich with the enzyme activity U defined by the vendor) to anticipate the trypsin activity of 60 U/mL after mixture with the diluted target enzyme samples. The intestinal-bicarbonate buffer solution with chymotrypsin at 20 U/mL was made by using the same intestinal-bicarbonate buffer solution above with the addition of 26 U/mL bovine chymotrypsin (0.0382 g of the bovine chymotrypsin powder per 50 mL of the buffer; 40 U/mg protein or 34,000.0 **U/g** of this C4129-500MG bovine chymotrypsin powder, Sigma/Aldrich with the enzyme activity U defined by the vendor) to anticipate the chymotrypsin activity of 20 U/mL after mixing with diluted target enzyme samples. The 1.25%-CMC substrate solution was made to contain 5 mM DTT with the 315-mOsm Krebs–Henseleit buffer. The 1.25%-CMC substrate solution was adjusted at pH 5.0 with the 2-M HCl solution to bring the final incubation mixture of the diluted enzyme preparation (0.050 mL); the intestinal-bicarbonate buffer (0.170 mL) and the 1.25%-CMC substrate solution (0.280 mL) at pH within 6.0–7.0 to allow the target enzymes to function in their optimal pH range^11,25^.

Target enzyme samples were each diluted fourfold by using the 315-mOsm Krebs–Henseleit buffer described previously. Diluted enzyme samples, the 1.25%-CMC substrate solution and the intestinal-bicarbonate buffer solutions (with trypsin at 60U/mL or chymotrypsin at 20 U/mL) were all pre-warmed in a 37 °C shaking water-bath for 30 min prior to be used for the designed experiments. All afore-mentioned buffers and solutions were thoroughly purged with ${\mathrm{N}}_{2}$ gas to deplete dissolved airborne O_2_ prior to be used for the designed experiments^42^.

Afterwards, 0.050 mL of the diluted enzyme preparations was mixed with 0.170 mL of the intestinal-bicarbonate buffer with trypsin (60 U/mL) or chymotrypsin (20 U/mL) to initiate the incubations. Immediately, the incubation samples’ Eppendorf tube headspace was purged with ${\mathrm{N}}_{2}$ gas and the samples were incubated in a shaking water-bath at 37 °C for their respective time periods of 0 (the positive control), 60, 90, 120, 180, 240 and 300 min, respectively. After incubation, each sample was then further mixed and incubated with 0.280 mL of the 1.25%-CMC substrate solution and the pH values of the samples were read and were ensured to be between the optimum pH range (6.0–7.0) of these target endocellulases^11,25^. The headspace of the samples’ tubes was purged again immediately with ${\mathrm{N}}_{2}$ gas and samples were incubated at 37 °C in a shaking water-bath for 15 min based on the previous linearity time course data obtained with the 1.25%-CMC substrate solution. To terminate the cellulases’ activity reaction incubations and begin the reducing sugar colour reaction, 1.500 mL of the DNS solution was added into each tube and the tubes were heated at 100 °C for 8 min on a heating block. Upon completion, samples were cooled down and reducing sugar absorbances were read on the microplate reader at 540 nm.

The incubation blank control group was also designed by mixing diluted enzyme preparations (0.050 mL) with intestinal-bicarbonate buffers (with trypsin at 60 U/mL) or chymotrypsin (at 20 U/mL) (each at 0.170 mL) for 300 min. Upon completion of the incubations, the blank control group was mixed with 0.280 mL of the 1.25%-CMC substrate solution and the mixture pH was recorded. Immediately after, 1.500 mL of the DNS solution was added and samples were mixed and heated and absorbances were read at 540 nm by using the BioTek Synergy H1 microplate reader (BioTek Winooski, VT, USA).

### Calculations and statistical analyses

Glucose-equivalent reducing products formed (µmol/mg protein) from the target enzyme reactions with using the CMC and/or Avicel substrates were calculated by using the absorbance values measured at 540 nm after correction for the sample blanks with a linear D-glucose calibration standard curve. Enzyme activity (nmol/mg protein•min) was further calculated by factoring glucose-equivalent product content with enzyme incubation time.

Plotting of the target enzymes’ activity [$v$, nmol/mg protein•min] against enzyme incubation time [t, min] was respectively best fitted with various polynomial regression models (linear, quadratic, cubic and quartic etc.); and exponential decay models. In order to mathematically partition the target enzymes’ activity inhibition responses of GH5-tCel5A1, the heat-treated GH5-tCel5A1 and GH5-p4818Cel5_2A, when examined under various in vitro physiological buffer conditions consisting of the gastric acidic pH (3.5), the gastric pH (3.5) with pepsin (274 U/mL) and the intestinal pancreatic trypsin (60 U/mL) and chymotrypsin (20 U/mL), the Eadie-Hofstee linear plot model was employed as shown in Eq. (1)^57^.

1 $$v={I}_{min}-{IC}_{50}*v/t$$

2 $${I}_{max}={I}_{c}-{I}_{min}$$

where $v$ is the enzyme activity (nmol/mg protein•min); ${I}_{min}$ is the minimal initial enzyme activity at the starting of enzyme incubations with a standard physiological buffer solution, i.e., the positive control group data (nmol/mg protein•min or % of the positive control, *I*_*c*_); ${IC}_{50}$ is the incubation time (h) with the in vitro treatment factors (the gastric pH, the gastric pH plus pepsin; the intestinal trypsin; and the intestinal chymotrypsin) at the half maximal inhibition (min); $t$ is the target enzyme incubation time (min) with the in vitro treatment factors; ${I}_{max}$ is the maximal enzyme activity due to inhibition by the in vitro treatment factors (nmol/mg protein•min or % of the positive control *I*_*c*_) calculated from Eq. (2)^57^; and ${I}_{c}$ is the mean initial target enzyme activity at the starting of enzyme incubations with the in vitro treatment factors (the positive control, nmol/mg protein•min or % of the positive control).

In order to calculate relative enzyme activity (% of the positive control) for the in vitro gastric-intestinal enzyme stability Experiment-1, target enzyme activities were compared to their initial enzyme activities measured at time-0 min (the positive control groups) of incubations with the various in vitro treatment factors after corrections for the sample blank contributions.

The exponential polynomials model; the Eadie-Hoftsee linear model; and the exponential decay model were all visualized and plotted using GRAPHPAD Prism (v8.3.0, GraphPad Software Inc., San Diego, CA, USA). Significance level at *P* < 0.05 was used for all the parameter estimates. All of the linear and non-linear curve analyses were done with the NLIN procedures in SAS (v9.4; SAS Institute Inc., Cary, NC, USA). Comparisons between two parameter estimates were carried out by the pooled two-tailed Student’s *t* test^58^.

## Abbreviations

Cys: Cysteine
CMC: Carboxymethyl cellulose
DNS: 3,5-Dinitrosalicyclic acid
DTT: Dithiothreitol
GH5: Glycoside hydrolase family-5
${IC}_{50}$: Half maximal inhibition incubation time
${I}_{c}$: Mean enzyme activity of control group
${I}_{max}$: Maximal magnitude of inhibition in the enzyme activity
${I}_{min}$: Minimal residual enzyme activity
IPTG: Isopropyl-β-D-thiogalactopyranoside
LB: Luria–Bertani
$${\mathrm{N}}_{2}$$: Nitrogen gas
OD: Optical density
